# Deciphering the Landscape of GATA-Mediated Transcriptional Regulation in Gastric Cancer

**DOI:** 10.3390/antiox13101267

**Published:** 2024-10-18

**Authors:** Rodiola Begolli, Anastasia Patouna, Periklis Vardakas, Anastasia Xagara, Kleanthi Apostolou, Demetrios Kouretas, Antonis Giakountis

**Affiliations:** 1 Laboratory of Molecular Biology and Genomics, Department of Biochemistry and Biotechnology, University of Thessaly, Biopolis, Mezourlo, 41500 Larissa, Greece; 2 Laboratory of Animal Physiology, Department of Biochemistry and Biotechnology, University of Thessaly, Biopolis, Mezourlo, 41500 Larissa, Greece; 3 Laboratory of Oncology, Faculty of Medicine, School of Health Sciences, University of Thessaly, Biopolis, Mezourlo, 41110 Larissa, Greece

**Keywords:** GATA4, GATA6, gastric cancer, AGS, MKN45, transcription, ChIP and RNA-seq, ROS, diagnosis, metagene signatures

## Abstract

Gastric cancer (GC) is an asymptomatic malignancy in early stages, with an invasive and cost-ineffective diagnostic toolbox that contributes to severe global mortality rates on an annual basis. Ectopic expression of the lineage survival transcription factors (LS-TFs) GATA4 and 6 promotes stomach oncogenesis. However, LS-TFs also govern important physiological roles, hindering their direct therapeutic targeting. Therefore, their downstream target genes are particularly interesting for developing cancer-specific molecular biomarkers or therapeutic agents. In this work, we couple inducible knockdown systems with chromatin immunoprecipitation and RNA-seq to thoroughly detect and characterize direct targets of GATA-mediated transcriptional regulation in gastric cancer cells. Our experimental and computational strategy provides evidence that both factors regulate the expression of several coding and non-coding RNAs that in turn mediate for their cancer-promoting phenotypes, including but not limited to cell cycle, apoptosis, ferroptosis, and oxidative stress response. Finally, the diagnostic and prognostic potential of four metagene signatures consisting of selected GATA4/6 target transcripts is evaluated in a multi-cancer panel of ~7000 biopsies from nineteen tumor types, revealing elevated specificity for gastrointestinal tumors. In conclusion, our integrated strategy uncovers the landscape of GATA-mediated coding and non-coding transcriptional regulation, providing insights regarding their molecular and clinical function in gastric cancer.

## 1. Introduction

Gastric cancer (GC) ranks fifth globally in both incidence and mortality [1]. With approximately 700,000 deaths and 1 million new cases recorded worldwide in 2022, GC accounted for around 7% of all cancer-related fatalities [1,2]. According to a 2022 study based on demographic projections, if current trends continue, the number of new cases is projected to globally increase by 62%, reaching 1.77 million by 2040 [3]. There is a considerable variation in GC incidence between sexes, with the incidence in males being twice as high as in females [1,4]. The disease’s global distribution fluctuates, with Eastern and Southeast Asia having a notably high incidence, followed by Latin America and Europe [5]. Approximately 70% of stomach cancers in the USA are of the intestinal subtype, which is the most prevalent form in high-incidence regions and whose development is preceded by molecular events collectively known as the Correa cascade [6,7]. In recent years, the incidence of GC has shown a steadily declining rate [8]. However, the mortality rates for this disease remain high, and there has been a noticeable rise in cases among younger people (less than 50 years old), despite the average age of disease onset being 72 years old [9,10,11,12]. The fact that many cases of the disease are detected at advanced stages, contributing to worse prognosis, is one of the main causes of the high fatality rates [13]. In particular, only 30% of patients with stomach cancer survive for longer than five years after diagnosis in the majority of affected countries [14]. The overall survival rate falls precipitously to 10% in cases involving advanced-stage diagnosis [15,16]. Currently, GC is diagnosed mainly via invasive and economically inefficient gastroscopic methods that are not applicable for routine monitoring of the general population [17]. Non-invasive diagnosis focuses on systemic biochemical biomarkers (e.g., CA19-9, CA72-4, CEA), pepsinogen on the mucous membrane of the stomach, and molecular biomarkers (e.g., HER-2, VEGF, CDH1), which lack both sensitivity and specificity [18,19]. As a result, survival prognosis of patients with advanced GC is poor, and the disease is frequently diagnosed at untreatable metastatic stages [13]. Therefore, development of new biomarkers involved in early stages of GC initiation is imperative. 

One of the primary factors that exacerbates the early diagnosis of GC is the strong molecular and cellular heterogeneity both within and between tumors, even in precancerous lesions [20,21]. The molecular classification of GC, along with numerous genome-wide studies, has revealed extensive genomic and epigenomic alterations occurring during the pathogenesis of the disease, such as aberrant DNA methylation and somatic copy number alterations (SCNAs) [22,23,24]. Advances in high-throughput technologies even at the single-cell level have made the detection of new driver oncogenes that function in a lineage-specific manner possible. These oncogenes are often referred to as lineage-survival oncogenes, with a central role in the theory of lineage-dependent cancer pathogenesis [25]. This model suggests that tumor cells heavily rely on survival mechanisms that are pre-programmed into lineage precursor cells during development and are influenced by acquired genetic alterations [25,26]. In contrast to oncogene addiction, which involves dependency on oncogenic gain/loss-of-function events, lineage addiction refers to the persistence and/or dysregulation of crucial lineage-survival pathways throughout the development of cancer or tumor formation [27]. Such lineage-survival factors are a distinct class of oncogenes. They are usually composed of transcriptional regulators of a particular cell lineage. When their expression or function is dysregulated, they aid in the growth and survival of tumors derived from that particular lineage [28]. One of the most representative examples is the master regulator MITF (microphthalmia-associated transcription factor), which is essential to melanocyte cell lineage differentiation [29]. In familiar or sporadic melanoma, MITF is overexpressed due to genomic amplification and acts as an oncogene by regulating downstream target genes such as *BCL2* (B-cell leukemia/lymphoma 2) and *CDK2* (Cyclin-dependent kinase 2), thereby affecting apoptosis and the cell cycle process [30,31,32]. Another interesting case of the lineage dependency concept in cancer is the process of the ectopic expression of master regulatory transcription factors that drive the trans-differentiation process of cell lineages [33]. An illustrative example is CDX2, a master regulator of the intestinal phenotype [34]. Its ectopic expression in the gastric mucosa drives the development of an intestinal cell lineage, leading to the neoplastic lesion of intestinal metaplasia, which is a precursor to GC [35,36]. 

Of particular interest are the master regulators GATA4 and GATA6 transcription factors, which are genomically amplified in GC [21,37]. The GATA transcription factor family is comprised of six members that play crucial, lineage-specific roles in regulating cell fate specification and differentiation across various tissues, thereby contributing to a wide range of physiological and pathological processes [38]. GATA transcription factors are DNA-binding proteins that share highly conserved two-zinc-finger domains [39]. These domains recognize the consensus DNA motif GATA, allowing the factors to bind to regulatory regions of the genome, interacting with other proteins to regulate transcription [40,41]. The GATA family members can be subdivided into two separate groups: GATA1/2/3, which are involved in hematopoietic and central nervous system cell differentiation, and GATA4/5/6, which are implicated in the differentiation of embryonic stem cells, cardiovascular embryogenesis, and adult epithelial cell differentiation [42]. Studies focusing on unraveling the developmental homeostasis of the stomach in mice revealed that *Gata4* and *Gata6* are involved in specifying the extraembryonic endoderm and are expressed in the foregut during endodermal regionalization, with *Gata4* playing a crucial role in the specification of the glandular stomach [43,44,45,46,47]. 

Aberrant expression of GATA4 and GATA6 contributes to cancer development, including GC, due to their critical role in physiological developmental processes [48]. At a chromatin level, GATA4 and GATA6 are considered pioneer factors that remodel closed chromatin to enable the binding of transcription factors and other co-factors, including themselves, to their target sites [49,50,51]. The functions of these transcription factors can vary, being either oncogenic or tumor-suppressive, depending on their interacting partners and the cellular context in which these interactions occur [52]. As a result, this type of master regulator may play different oncogenic or tumor-suppressive roles in various types of cancer. For example, GATA4 has a tumor-suppressive effect in lung cancer, where it is not normally expressed. Ectopic expression of GATA4 in mice has been shown to affect the TGF-β2 signaling pathway and induce cell senescence [53]. In contrast, GATA4 also exhibits an oncogenic effect in nasopharyngeal cancer, where it promotes cell proliferation and epithelial–mesenchymal transition [54]. Similarly, research on GATA6 has revealed its oncogenic role in non-small cell lung cancer (NSCLC) by reducing cell autophagy and cell viability in the H1650 cell line, while in lung cancer it inhibits cell growth in vitro and tumorigenesis in vivo [55,56]. Conversely, GATA6 plays a tumor-suppressive role in cholangiocarcinoma, promoting epithelial–mesenchymal transition (EMT) and metastasis through the MUC1/β-catenin pathway [57].

This controversial function of master regulators like GATA4 and GATA6 in cancer, alongside their crucial functions in maintaining normal tissue balance, complicates efforts for their direct utilization in diagnosis or treatment. This has led to a focus on their downstream targets, particularly in GC, where the research on the function and regulatory capacity of these transcription factors is limited. Of particular interest is the detection of non-coding RNAs regulated by lineage-survival transcription factors, as this could increase the tissue- and cancer-specific utility of these targets for potential diagnostic and therapeutic applications in the future.

## 2. Materials and Methods

### 2.1. Cell Acquisition and Culture

The GC cell lines AGS and MKN45 were a kind gift from Prof. Dionyssios Sgouras from the Greek Institute of Pasteur, Athens, Greece. Both lines were cultured in RPMI Gibco (London, UK) containing 10% (*v*/*v*) fetal bovine serum, 2 mM L-glutamine (Gibco, UK), 100 units/mL of penicillin, and 100 units/mL of streptomycin (Gibco, UK) in plastic disposable tissue culture flasks at 37 °C in 5% CO_2_. Each line was periodically checked every month for mycoplasma contamination. 

### 2.2. Cell Transfection and Generation of Stable Cell Lines

The stable AGS cell lines with inducible knockdown of GATA4 or 6 transcription factors or with scrambled shRNAs were generated in two stages with the pTER system [58]. First, a lentiviral transduction system was used to create an AGS cell line with stable overexpression of the Tetracycline Repressor (TetR) protein. Lentiviral particles were produced in HEK-293 cells using the following plasmids: pLenti CMV TetR Blast (716-1) (Addgene, plasmid #17492, RRID, (Watertown, MA, USA)), psPAX2 (Addgene plasmid #12260, RRID), and pMD2.G (Addgene plasmid #12259, RRID). Genetic modification was carried out using the Jetprime (Polyplus, #101000046, (Illkirch-Graffenstaden, France)) transfection reagent following the manufacturer’s instructions. AGS cells (2.5 × 106) were incubated with 10 mL of viral medium at a 0.5× dilution and 160 μL of Polybrene transfection reagent (Millipore (St. Louis, MO, USA), #TR-1003-G) for 24 h. The medium was refreshed, and the cells were allowed to recover overnight. At 16 h after virus removal, selection medium was added with 1 μg/mL of Blasticidin S (ChemCruz,#sc-204655, (TE Huissen, The Netherlands)). After 14 days, surviving clones were mechanically selected for clonal expansion, during which they were maintained in the same selection medium. Clone screening was performed via qPCR to detect TetR expression. The clone with the highest TetR expression underwent further genetic modification using Polyethylenimine (Polysciences Inc. (Warrington, PA, USA)) as a transfection reagent to introduce a PTER+ construct for shRNA expression along with a Zeocin resistance gene. The shRNAs of interest were synthesized (Eurofins, Ebersberg, Germany) and individually cloned into the PTER+ plasmid [59] downstream of a TetR-binding motif, creating cassettes that enable doxycycline-inducible shRNA expression. After the transfection of 1 × 10^6^ cells with 5 ug linearized of each plasmid and 3:1 DNA to Polyethylenimine ratio, clone selection was conducted using two antibiotics (Zeocin and Blasticidin) (Zeocin, Invitrogen (Carlsbad, CA, USA), #R25001) for 14 days. Surviving clones were mechanically selected, clonally expanded in a dual selection medium containing 1 μg/mL of Blasticidin and 0.5 mg/mL of Zeocin and tested for GATA4 or GATA6 expression levels by qPCR with and without 1 ng/μL doxycycline to induce silencing. The lentiviral transduction system was also utilized to transfect MKN45 cells with the pSicoR plasmid (Addgene plasmid #12084, [60]), which is a lentiviral compatible vector suitable for shRNA expression. To induce shRNA expression in MKN45 cells, 2.5 × 10^5^ cells per transgene were incubated with the virus medium along with the polybrene transfection reagent (as previously described) for 24 h. Knockdown efficiency was evaluated with qPCR and Western blot 48 h after the virus was removed. All shRNA sequences are listed in Table 1.

### 2.3. Western Blotting

For the Western blot analysis, following knockdown induction for three days, cells were washed twice with PBS and collected into a RIPA buffer containing 50 mM Tris pH7.5, 1% NP40, 0.25% Na-Deoxycholate, 150 mM NaCl, 1 mM EDTA, and 10% glycerol. The protein concentration was measured spectrophotometrically using the Q3000 spectrophotometer (Quawell, San Jose, CA, USA). An equal volume of 2× SDS lysis buffer (100 mM Tris, pH 6.8, 4% SDS, 10% glycerol, 2% 2-mercaptoethanol) was added, and the protein samples were separated by SDS-PAGE. The proteins were transferred onto a nitrocellulose membrane (Macherey-Nagel, Porablot NCP, #741280, (Dueren, Germany)) and, after blocking with 5% skim milk in TBST for 1 h at RT, were incubated with primary antibodies (Santa Cruz,#sc-25310, #sc-518050, (Heidelberg, Germany) and Cell signaling, #8H10D10,(Danvers, MA, USA)) at a 1:1000 dilution overnight. The membranes were then washed four times with TBST and the HRP-conjugated secondary antibody (cell signaling) was added at a dilution of 1:10,000 in TBST and incubated for 1 h. Finally, the membrane was washed again four times with TBST, and the ECL Prime Western blotting Detection Reagent kit (Amersham, #RPN 2232, (Slough, Buckinghamshire, UK)) was used for the visualization of protein bands with Kodak film exposure.

### 2.4. Colony Formation Assay

AGS cells (500 cells/well) were cultured in 6-well plates for 14 days under two experimental conditions: the knockdown condition (KD), where doxycycline was added to the medium to induce shRNA expression, and the control condition, with cells grown in a normal medium. A fresh medium containing doxycycline (TAKARA, #631311, (Saint-Germain-en-Laye, France)) at a final concentration of 1 ng/μL was replenished every 4 days after seeding. After 14 days of incubation, the colonies were washed with PBS and then stained with 1% Methylene Blue (Sigma-Aldrich #M9140 (St. Louis, MO, USA)) solution for 5 min. Subsequent washes with distilled water were performed to remove background staining. MKN45 cells (1.5 × 10^5^ cells/well) were also cultured in 6-well plates. Knockdown was induced via virus incubation for 24 h, and 48 h later, the cells were stained. The stained colonies were photographed using a digital camera, and their number was quantified using ImageJ software (v2). Statistical analysis was performed with the data analysis toolkit in Excel and represents *t*-test results with equal variance. 

### 2.5. Flow Cytometry Analysis for Cell Cycle

After knockdown induction for three days, 2 × 10^6^ cells were washed twice with PBS and resuspended in 2 mL of PBS. Next, 5 mL of ice-cold 70% ethanol and 0.5 mL of PBS (0.1% glucose) were added for overnight fixation at 4 °C. Following fixation, cells were centrifuged (5 min/1500 rpm/4 °C) and washed twice with 10 mL of PBS. The cells were then pelleted and incubated in a staining solution containing 0.4 mg/mL propidium iodide (PI) and 40 μg/mL RNase A (GeneOn BioScience, #409-050 (Ludwigshafen, Germany)) for 40 min at room temperature in the dark. Stained nuclei were analyzed by flow cytometry using the FACS Melody instrument (BD Biosciences (Franklin Lakes, NJ, USA)) with the PE channel for PI detection. FCS files were analyzed using FlowJo software v10.10, and statistical analysis was performed using ANOVA (Holm-Sidak) in SigmaPlot v10.

### 2.6. FACS Analysis for Apoptosis

After knockdown induction, apoptosis analysis was performed using the FITC Annexin V Apoptosis Detection Kit I (BD Pharmingen, #556547, (Heidelberg, Germany)) following the manufacturer’s instructions. Briefly, 1 × 10^6^ cells were washed twice with 2 mL of PBS and centrifuged (5 min/1500 rpm/4 °C), and the pellet was resuspended in 100 μL of binding buffer. FITC Annexin V and propidium iodide (PI) were then added, and the cells were incubated for 15 min at room temperature in the dark. Subsequently, 400 μL of binding buffer was added, and flow cytometry was performed. The FACS Melody instrument (BD Biosciences) was used with the FITC channel for Annexin V detection and the PE channel for PI detection. FCS files were analyzed using FlowJo software, and statistical analysis was performed using ANOVA (Holm-Sidak) in SigmaPlot.

### 2.7. Wound Healing Assay 

AGS and MKN45 cells (5 × 10^5^ cells/well) were cultured in 6-well plates under similar experimental conditions (control and KD) as those described for the colony formation assay. After 24 h of cell seeding, cell culture reached confluence, and a scratch was made in the center of each well using a pipette tip. The medium containing doxycycline at a final concentration of 1 ng/μL was refreshed every 24 h. Images were taken at 0, 12, 24, 36, and 48 h after the scratch, and the wound area was analyzed using ImageJ software. All experiments were conducted in triplicate, with three fields counted per well. Statistical analysis was performed using the data analysis toolkit in Excel, and the results represent *t*-test outcomes with equal variance.

### 2.8. FACS Analysis for Determining Reduced Glutathione (GSH) and Reactive Oxygen Species (ROS) Levels

AGS and MKN45 GC cells were cultured in 12-well plates at 37 °C in 5% CO_2_ atmosphere and grown to 70–80% confluency. To determine GSH levels, the culture medium was discarded, and the cells were washed with ice-cold phosphate-buffered saline (PBS) (Gibco, UK), trypsinized, and centrifuged (1000 rpm, 5 min, 4 °C). The supernatant was discarded, and the cell pellets were resuspended in ice-cold PBS and centrifuged (1000 rpm, 5 min, 4 °C). Then, the supernatant was discarded, and the cell pellets were resuspended in 1 mL ice-cold PBS and 5 μL Thiol Green Dye (200X) (Abcam, Cambridge, UK), dissolved in dimethyl sulfoxide (DMSO) (Abcam, UK), and incubated in the dark at 37 °C for 30 min. After incubation, the cells were centrifuged (1000 rpm, 5 min, 4 °C), the supernatant was discarded, and the cell pellets were resuspended in ice-cold PBS and subjected to flow cytometry using the FL1 channel to monitor the fluorescence intensity. To determine ROS levels, the culture medium was discarded, and the cells were rinsed with ice-cold PBS, trypsinized, and centrifuged (1000 rpm, 5 min, 4 °C). The supernatant was discarded, and the cell pellets were resuspended in ice-cold PBS and centrifuged (1000 rpm, 5 min, 4 °C). Then, the supernatant was discarded, and the cell pellets were resuspended in 1.5 mL 2′,7′-dichlorodihydrofluorescein diacetate (H_2_DCFDA) (10 μΜ) (Gibco, UK), dissolved in PBS, and incubated in the dark at 37 °C for 45 min. After incubation, the cells were centrifuged (1000 rpm, 5 min, 4 °C), the supernatant was discarded, and the cell pellets were resuspended in ice-cold PBS and subjected to flow cytometry using the FL1 channel to monitor the fluorescence intensity. Flow cytometry was performed with the BD FACSCalibur^TM^ instrument (BD Biosciences) using the BD Cell Quest Pro software v 6.0 (BD Biosciences) for acquiring and analyzing data. The event rate was ≈300 events/s, and a total of 10,000 events were collected for each sample. The fluorescence intensities were measured on a logarithmic scale. Statistical analysis was performed with the data analysis toolkit in Excel and represents *t*-test results with equal variance.

### 2.9. Spectrophotometric Analysis for Determining Thiobarbituric Acid Reactive Substances (TBARS) Levels

AGS and MKN45 GC cells were cultured in 6-well plates at 37 °C in 5% CO_2_ atmosphere and grown to 70–80% confluency. Then, the culture medium was discarded, and the cells were washed with ice-cold PBS, trypsinized, and centrifuged (1000 rpm, 5 min, 4 °C). The supernatant was discarded, and the cells were washed with ice-cold PBS and centrifuged (1000 rpm, 5 min, 4 °C). The supernatant was discarded, and the cell pellets were resuspended in ice-cold PBS containing a protease inhibitor cocktail tablet (Roche Diagnostics, Mannheim, Germany) and subjected to periodical sonication for 50 s on ice using an ultrasonic processor (UP400S, Hielscher Co. (Teltow, Germany)). After that, the cells were centrifuged (15,000× *g*, 20 min, 4 °C), and the supernatant, i.e., the cell lysate, was collected. The total protein concentration was estimated by performing the Bradford assay and using a standard curve of bovine serum albumin (BSA) (Sigma-Aldrich, Steinheim, Germany). The TBARS levels were determined on the basis of the experimental protocol of Keles et al. [61]. In particular, cell lysates containing 50 μg of total protein were mixed with 500 μL Tris-HCl buffer (200 mM, pH = 7.4) and 500 μL 35% trichloroacetic acid (TCA) (Sigma-Aldrich, Germany) and incubated for 10 min at room temperature. Then, 1 mL of a sodium sulfate (Na_2_SO_4_) (2 M) (Sigma-Aldrich, Germany)—thiobarbituric acid (TBA) (55 mM) (SERVA, Germany) solution was added, and the samples were vortexed and placed in a water bath for 45 min at 95 °C. Subsequently, the samples were transferred on ice for 5 min, 1 mL 70% TCA was added, and the samples were centrifuged (11,200× *g*, 3 min, 25 °C). The optical density was measured at 530 nm with a UV–vis spectrophotometer (UV-1600PC, VWR). To calculate TBARS concentration, we used the molar extinction coefficient of malondialdehyde (ΜDA) (156,000 L/mol/cm). Statistical analysis was performed with the data analysis toolkit in Excel and represents *t*-test results with equal variance.

### 2.10. ChIP-Seq Data Analysis

The raw FASTQ files from accession numbers PRJNA224585 and GSE85467 were downloaded from GEO and subjected to FASTQC analysis. Subsequently, the FASTQ files were aligned against the human genome (hg19) with MACS [62]. Peak annotation was performed with ChIPSeeker [63], and heatmap construction upset plots and Transcriptional Start Site (TSS) distribution plots were performed with Clusterprofiler [64] in R. Motif enrichment analysis was performed with the Docker version of the Sea package. Relative peak distance analysis was performed with the reldist algorithm of the bedtools suit in Linux. Density plots were created with custom ggplot2 code in R. Relative peak enrichment plots were created with ggplo2 in R. Functional enrichment analysis was performed with DOSE in R. All dot plots were created with the custom ggplot2 code in R.

### 2.11. NGS Sequencing

Total RNA was isolated from AGS cells following inducible GATA4 and 6 knock down for three days with the TRI reagent (#TR-118, MRC). The isolated RNA was subjected to quality control and quantification with gel electrophoresis and a Quawell spectrophotometer (Q3000, Quawell). QC and quantification were repeated with Bioanalyzer prior to Illumina library construction, as described previously [65]. In brief, next generation sequencing was performed with the Illumina Nova seq platform, resulting in at least 20M reads per sample. FASTQ results were subjected to quality check with FASTQC and subsequently to genome alignment with HISAT2 against hg19. Subsequent statistical analysis of all RNA-seq data were submitted to GEO under the accession number GSE274529. 

### 2.12. RNA-Seq Analysis

Heatmap analysis was performed with pheatmap in R. Deregulogram was performed with ggplot2 based on the RNA-seq results. Venn diagrams were created with custom R code. The regulatory score analysis was performed with the TIP algorithm [66] in R. GSEA and functional enrichment analyses were performed with DOSE in R, and dot plots were created with ggplot2.

### 2.13. cDNA Synthesis and RT-qPCR Analysis

Total RNA was extracted by the TRIzol extraction technique (MRC, #TR-118). The extracted RNA was then DNAse treated (Thermofischer, #EN0521, Waltham, MA USA) by incubating at 37 °C for 1 h, followed by phenol/chloroform extraction and ethanol precipitation. The DNAse-treated RNA was quantified spectrophotometrically using the Quawell Q3000 spectrophotometer. Then, 1 μg of DNAse-treated RNA was used for first-strand cDNA synthesis (Thermofischer #K1612) and was primed with random hexamers (TAKARA, #3802) according to the manufacturer’s instructions. The cDNA was diluted in 400 μL of ddH2O, and 4 μL was used for RT-qPCR reactions using KAPA SYBRGREEN qPCR mix (KAPA Biosystems # KK4618, Wilmington, MA, USA). The qPCR reactions were performed in duplicate, and the primers used can be found in Table 2. qPCR was performed in a BIORAD CFX96 qPCR machine under the following conditions: 95 °C for 3 min, followed by 44 cycles of 95 °C for 10 s, 58 °C for 20 s, and 72 °C for 30 s, with a final step at 72 °C for 2 min. A melting curve was generated by ramping from 55.0 °C to 95 °C at an increment of 0.5 °C for 0:05. The 2^−Δct^ value was calculated with Excel 365 and normalized with GAPDH housekeeping values to determine the fold difference between the controls and the knockdown samples.

### 2.14. The Cancer Genome Atlas (TCGA) and Genotype to Expression (gTEX) Data Analysis

RNA-seq from gastric tumors and clinical information from the respective patients were downloaded from TCGA with the GDC client (dbGAP access approval TCGA #117269-1). Normal gene expression for gastric biopsies was retrieved from gTEX (DUOS gTEX access approval DUOS-DAR #350). Expression matrixes were cross-normalized, log transformed, and converted to z-scores. A heatmap was created with the pheatmap package using the associated clinical information for the tumor samples from TCGA for annotation purposes. ROC analysis was performed as described previously [67], and bee-swarm and violin plots were created with ggplot2 in R. Boxplot gastrointestinal analysis and plots were created with the GEPIA platform. Kaplan–Meier analysis was performed with the survminer (version 0.4.9) package in R. 

## 3. Results

### 3.1. GATA4 and 6 Expression in Cancer Patients

**Figure 1:**
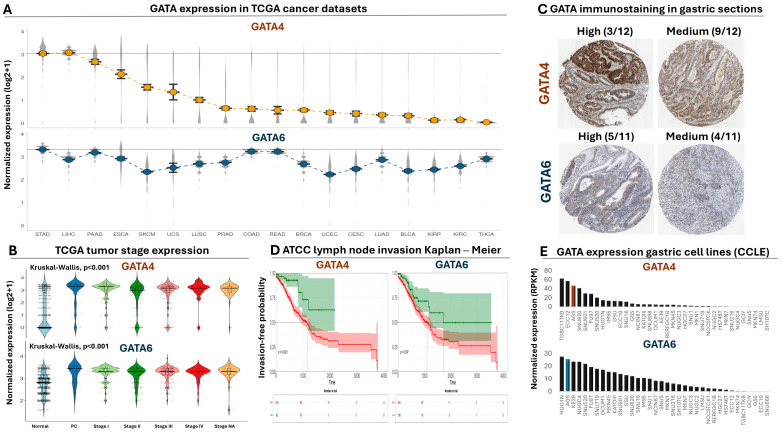
Analysis of GATA4 and 6 expression in cancer patients. (**A**) Normalized expression of GATA4 (upper plot) and GATA6 (lower plot) in RNA-seq data from a multi-cancer panel of TCGA tumors. Dots correspond to average expression and grey density plots to its distribution for each cancer type. Cancer types are aligned from left to right according to decreasing levels of GATA4 expression for both plots. (**B**) Violin plots comparing the expression of GATA4 (upper plot) and GATA6 (lower plot) in staged gastroesophageal tumors from TCGA compared to normal biopsies. (**C**) Representative immunostaining images of GATA4 and GATA6 protein levels in gastric tumors (1 mm) from the Human Protein Atlas. The number of tissue sections with high or medium expression is shown above each image compared to the total. None of the available sections had low or non-detected levels of GATA4, while only 2 out of 11 sections had low/not-detected levels of GATA6. (**D**) Kaplan–Meier analysis for comparing lymph node invasion between patients with high (red curve) and low (green curve) levels of GATA4 (left plot, log2rank = 0.021) or GATA6 (right plot, log2rank = 0.007). (**E**) Bar plot demonstrating the expression of GATA4 (upper plot) or GATA6 (lower plot) from RNA-seq experiments in all available gastric cancer cell lines from the Cancer Cell Line Encyclopedia. Expression of both factors in AGS gastric cancer cells is marked with orange or blue, and cell lines are ranked from left to right according to decreasing levels of GATA expression.

Analysis of RNA-seq data from the TCGA consortium revealed that both the *GATA4* and *GATA6* transcripts are on average expressed at significantly higher levels in gastric tumors compared to a collection of solid cancer types (Figure 1A), with the exception of hepatocellular carcinoma tumors, in which *GATA4* levels were not significantly different compared to gastric tumors (Supplementary Table S1). Moreover, comparison of *GATA4* and *6* expression in staged gastroesophageal tumors revealed statistically significant upregulation for both transcripts compared to normal biopsies (Figure 1B, Supplementary Table S2), an observation that is consistent with the elevated levels of both GATA4 and 6 proteins in gastric tumors based on the Human Protein Atlas (Figure 1C). We also observed a statistically significant inverse correlation between high levels of *GATA4* or *6* and invasion-free probability in gastric tumors (Figure 1D). Of note, expression of both transcription factors is evident in the majority of the commercially available GC cell lines (Figure 1E). Taken together, these data confirm the significant upregulation of both transcription factors in gastric tumors and support their prognostic impact on the disease. 

### 3.2. GATA ChIP-Seq Analysis in AGS GC Cells

Given the marked presence of GATA4 and 6 in gastric tumors, a collection of publicly available ChIP-seq data (see M&M) was used in order to molecularly dissect their regulatory role in the disease, focusing on their chromatin interactions in GC cells. This collection consists of GATA4 and 6 immunoprecipitations along with chromatin modifications that are typically associated with regulatory DNA, namely H_3_K_27_ac (chromatin accessibility), H_3_K_4_me3 (active promoters), and H_3_K_4_me1 (enhancers). We focused our analysis on the AGS GC cell line, since these cells were among the top expressing lines both for GATA4 and GATA6 (Figure 1E), they were included in the ChIP-seq collection and are available in our laboratory, facilitating downstream experiments. Genomic alignment yielded 165.398 and 120.414 unfiltered peaks for GATA4 and 6, respectively, which were subjected to peak annotation and statistical metanalysis. 

#### 3.2.1. Genomic Distribution of GATA4 and 6 Binding 

**Figure 2:**
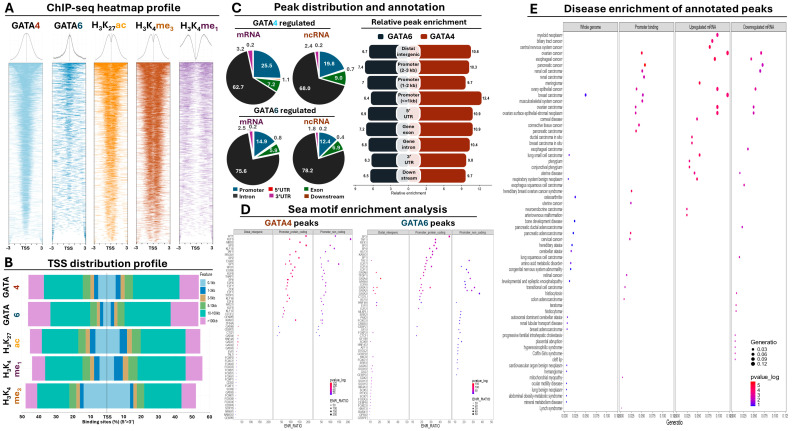
ChIP-seq analysis of GATA4 and 6 along with histone modifications in AGS cells. (**A**) Global heatmap analysis of ChIP-seq peak distribution around the TSS. (**B**) TSS distribution plot summarizing the percentage of peaks across all genomic annotations. (**C**) GATA4 and 6 peak distribution across genic annotations (left panel) and relative peak enrichment analysis over background for all genomic annotations. (**D**) Sea motif enrichment analysis for GATA4 and 6 peaks divided for all peaks (left), protein-coding promoters (middle), and non-coding promoters. Color indicates statistical significance; dot size indicates enrichment score. (**E**) Dot plot summarizing the disease enrichment results from the annotated ChIP-seq peaks. First panel on the left corresponds to results from all GATA peaks, the second panel corresponds to promoter peaks only, the third panel to the promoter peaks of all GATA upregulated genes, and the fourth panel to the promoter peaks of all GATA downregulated genes. Color indicates statistical significance; dot size indicates gene ratio for each category.

Peak annotation of GATA4 and 6 ChIP-seq data revealed the presence of these transcription factors upstream of both protein-coding and non-coding genes in AGS GC cells (Figure 2A). However, the distribution of GATA peaks was predominantly shifted towards distal intergenic regions (Figure 2B). More specifically, 20% of all GATA4 peaks resided within a maximum distance of 5 Kb around the TSS of genes. 

This promoter-associated fraction consists of 10.3% of total peaks residing up to 1 Kb, 5.3% residing between 1 and 2 Kb, and 4.4% residing between 3 and 5 Kb around the TSS. It should be noted that these percentages do not represent GATA peaks found exclusively upstream of the TSS, suggesting that in some cases, GATA presence follows an intragenic binding pattern (Supplementary Figure S1A). Another 9.5% of GATA4 peaks were located between 5 and 10 Kb from the TSS, yet the vast majority (50%) resided at distal intergenic regions spanning between 10 and 100 Kb for the TSS. Finally, the remaining 20.4% of GATA4 peaks resided at distances that exceeded 100 Kb from the TSS (Figure 2B). 

In comparison, GATA6 binding was even more associated with a distal intergenic binding pattern. The overall promoter presence of this transcription factor dropped to 12% (consisting of 4.5% up to 1 Kb, 4% between 1 and 3 Kb, and 3.5% between 3 and 5 Kb), with another 8% residing between 5 and 10 Kb away from the TSS. These percentages are even more striking considering that their sum is considerably less than the overall promoter presence of H_3_K_4_me1 (22.3%), a histone mark that is traditionally associated with distal intergenic regions rather than proximal promoter sites. Similar to GATA4, the percentage of all GATA6 peaks residing between 10 and 100 Kb was equal to 50%, with the remaining 30% representing true intergenic binding localized at distances that exceeded 100 Kb from the TSS of genes (Figure 2B). In conclusion, both transcription factors reside around promoters in AGS GC cells, yet the majority of their chromatin interactions occur in distal intergenic regions, also enriched for the H_3_K_27_ac and H_3_K_4_me1 histone marks that decorate enhancer elements. It should be noted, however, that the observed distal intergenic enrichment of the GATA peaks can be attributed in part to the overall nucleotide length of the associated regions, which is on average larger compared to proximal TSS annotations.

Focusing exclusively on the genic annotations, we observed that the distribution of GATA4 peaks had a small preference for binding to the promoter of protein-coding genes compared to the remaining annotations (Figure 2C, Supplementary Table S3). This promoter preference was slightly reduced in ncRNA genes, in which a small increase for intron binding was observed. GATA6 peaks generally agreed with this pattern, although the promoter preference was overall reduced both for protein-coding and non-coding genes compared to GATA4 (Figure 2C). Beyond mere genomic distribution, certain conclusions can be drawn when the relative enrichment (representing binding strength) of GATA binding across different genomic annotations was examined. For example, overall enrichment of GATA4 binding was on average stronger compared to GATA6 across all examined genomic annotations (Figure 2C). When the analysis focused on the evenly spaced promoter annotations, GATA4 binding was particularly enriched at the chromatin that surrounds the TSS (0–1 Kb) and became progressively reduced in intragenic or distal intergenic regions. In contrast, relative enrichment of GATA6 binding was more or less evenly distributed across all genomic annotations (Figure 2C). In conclusion, these observations suggest that strong GATA4 binding occurs more frequently at the proximal promoter region of protein-coding and non-coding genes compared to the more evenly distributed pattern of GATA6 binding. 

We also subjected GATA4 and 6 peaks along with H_3_K_4_me3 and H_3_K_4_me1 to a relative peak distance analysis, designed to provide insights regarding their co-existence across multiple genomic locations. As expected, GATA4 and GATA6 peaks overlapped with each other; however, their co-occurrence was more pronounced at distal intergenic regions compared to proximal promoters (Supplementary Figure S1B). Importantly, their combinatorial intergenic binding also overlapped with H_3_K_4_me1, overall suggesting that both lineage survival transcription factors reside at intergenic enhancer elements in part to facilitate their downstream regulatory functions. Focusing on the TSS, GATA4 peaks strongly overlapped with H_3_K_4_me3 signals, especially at the proximal promoter chromatin located 0–1 Kb around the TSS. Their close association persisted in more distal promoter locations (1–2 and 2–3 Kb around the TSS), albeit with a small yet observable decline both in terms of density (strength) and uniformity (distribution). A similar trend was reflected also for GATA6 binding, with a slightly reduced probability for an overlap with H_3_K_4_me3 at proximal promoter chromatin compared to GATA4 (Supplementary Figure S1B). Collectively, these observations suggest that both transcription factors co-exist at the chromatin of enhancers and promoters, confirming their strong regulatory role in GC cells. 

#### 3.2.2. DNA Motif Enrichment Analysis of GATA Peaks

Having established the presence of both GATA factors at the chromatin of promoters and enhancers, we focused next on the analysis of the DNA sequence from their peaks with the aim of identifying enriched motifs of other transcription factors and chromatin-associated proteins that co-localize around the GATA motif at proximal and distal regulatory elements. Motif enrichment was performed separately for distal intergenic as well as promoter peaks of protein-coding or non-coding genes, providing detailed insights regarding the co-occurrence of the GATA motif with other factors across these genomic annotations (Figure 2D, Supplementary Table S4). 

Regarding the GATA4 peaks in distal intergenic regions, apart from the expected co-occurrence of various GATA motifs, we observed enrichment of various members from the FOX family of transcription factors, known for their involvement in gut development and GC. Interestingly, we also observed enrichment for the HNF4A and CDX2 motifs, which cooperatively bind enhancer elements to promote GC through intestinal metastasis, along with enrichment of CENPA and B, which control centromeric chromatin during the S and G2/M phases of cell cycle. Importantly, the repertoire of motifs that co-reside with GATA4 at promoter elements was accompanied by overall higher enrichment scores and was qualitatively different from that at distal intergenic regions (Figure 2D). For example, we frequently observed co-enrichment of various E2F members at the promoter of both protein-coding and non-coding genes, suggesting that GATA4 shares part of its downstream targets with the transcriptional output of the E2F family that is also involved in the regulation of cell cycle checkpoints. We also confirmed co-occurrence of KLF motifs known to interact with GATA members to control gastrointestinal (GI) development and carcinogenesis. Unexpectedly, we observed enrichment of the CTCF and CTCFL motifs at the promoters of both coding and non-coding genes, pointing towards chromatin interactions with distal elements, yet the same motifs were not enriched at the distal intergenic regions of GATA4 peaks (Figure 2D, Supplementary Table S4).

Motif enrichment analysis of the GATA6 peaks was in broad agreement with the GATA4 observations, yet with marked differences both at distal intergenic and promoter regions. For example, we did not observe a pronounced enrichment of FOX family members for GATA6 peaks at distal intergenic peaks, but instead various SOX family motifs were enriched. Similar to the GATA4 results, enrichment of HNF4A and CDX2 motifs was observed (Figure 2D, Supplementary Table S4). At promoter regions, we observed enrichment of KLF, E2F, and CEBP motifs, along with CTCF/CTCFL, confirming GATA4 observations. Interestingly, unlike GATA4 that shared the enrichment of common motifs between coding and non-coding promoter peaks, in GATA6 peaks, we observed several motifs that were uniquely enriched only at the latter (Figure 2D, Supplementary Table S4). These motifs included but were not limited to the steroid hormone receptors ERR2 and 3, members of the PAX family, CREB5, and ATF1. Most of these proteins are associated with embryonic development and/or cancer-related processes, such as cell proliferation and metastasis, collectively suggesting that GATA-mediated regulation of non-coding RNAs could be involved in the regulation of physiological and pathological processes in the gastrointestinal tract and beyond. 

#### 3.2.3. Functional Analysis of GATA Annotated Peaks 

Having established a regulatory role for the observed GATA chromatin interactions, we subsequently focused on the functional and disease enrichment analysis of their annotated ChIP peaks. We performed this analysis in two interconnected axes. The first axis referred to the analysis of the associated genes from all ChIP peak annotations (referred to as whole genome thereof) or genes for which GATA presence was restricted only at the promoter (referred to as promoter binding) (Figure 2E). Therefore, this analytical axis served as a general overview of GATA-associated functions. However, to gain direct insights regarding the GATA-mediated regulation in GC cells, we took advantage of the RNA-seq data from our inducible GATA knockdown lines in AGS cells (see below), subjecting all up- and downregulated mRNAs with GATA4 and/or 6 peaks around their TSS to functional/disease enrichment analysis (Figure 2E). 

According to the results from the disease enrichment analysis, a close association of GATA chromatin interactions with genes that are involved in various pathologies (Figure 2E, Supplementary Table S5) was confirmed. More specifically, whole genome peak annotations were mostly enriched in non-cancerous diseases, while promoter annotated peaks not only had higher *p*-values on average but were predominantly enriched in various forms of cancer, including malignancies of the GI or the reproductive system. Importantly, analysis of both up- and downregulated genes with GATA promoter peaks confirmed the previous results, revealing even stronger statistical associations with carcinogenesis, especially for GI tumors (e.g., esophageal and biliary track neoplasms) or the central nervous system. Taken together, these data firmly establish the role of GATA4 and 6 chromatin interactions with the direct regulation of genes involved in various forms of cancer, including but not limited to GI carcinogenesis. 

Beyond disease associations, we repeated the same analysis for functional associations in the form of reactome pathway enrichment (Supplementary Figure S1C, Table S5). Examination of whole genome annotations revealed statistical association of GATA presence with cellular responses to oxidative stress, epithelial morphogenesis, organ development, and stem cell differentiation, among other categories. Instead, genes with promoter-associated peaks were enriched for phospholipid metabolism, response to cellular stress, DNA replication, and mitochondrial organization. Importantly, analysis of the differentially expressed subset of genes with promoter peaks revealed strong associations with apoptosis, wound healing, keratinocyte differentiation, and response to glucose starvation, all of which were specific to the upregulated genes. Instead, investigation of the downregulated genes with promoter peaks revealed functional enrichment for the regulation of cell cycle, mitotic transitioning, sister chromatin segregation, and sterol metabolism (Supplementary Figure S1C, Table S5). Collectively, these functional categories not only complement the observed enrichment of GATA-mediated regulation in carcinogenesis (Figure 2E) but also provide a mechanistic basis for the observed phenotypes that accompany impairment of GATA-mediated regulation in GC cells (see below).

### 3.3. Transcriptome Analysis of Inducible GATA4 and 6 Knockdown in AGS GC Cells 

As shown above, analysis of ChIP-seq data can provide valuable insights regarding the regulatory potential and/or functional role of GATA4 and 6 in GC cells. However, since transcription factor binding to the chromatin is not always equivalent with transcriptional regulation, we decided to utilize an inducible, shRNA-mediated knockdown (KD) approach against GATA4 and 6 coupled to transcriptome analysis to gain transcriptional insights regarding the regulatory effect of both factors on the transcription of coding and non-coding genes in GC cells. For the previously explained reasons, our experimental design focused on AGS cells, engineered to express the tetracycline repressor protein that in turn controls the expression of small hairpin RNAs targeting either GATA4, GATA6, or a scrambled sequence. This inducible system incorporated a TetR binding motif upstream of the shRNA region, allowing the TetR protein to suppress the activity of the U6 promoter. Adding doxycycline impeded TetR binding, enabling shRNA expression and subsequently knocking down the target transcription factors, a result which we confirmed with RT-qPCR and Western blot analysis (Supplementary Figure S2A,B, Table S6). 

#### 3.3.1. Identification of Differentially Expressed Genes for GATA KD

Differential expression analysis of RNA-seq data revealed extensive changes in the transcriptome of AGS GC cells after inducible KD of GATA4. More specifically, we detected 7.021 differentially expressed genes (DEGs), corresponding to ~12% of the total transcriptome annotations. These DEGs were further subdivided to 3.851 (or 54.8%) significantly upregulated and 3.170 (or 45.1%) significantly downregulated genes, revealing a slight imbalance towards upregulation. In terms of biotype representation, 5.477 genes corresponding to 78% of all DEGs were protein-coding, a ratio that was maintained in the upregulated and downregulated DEGs. The number of DEGs in the affected transcriptome of GATA6 KD were slightly reduced (6.713 or ~11% of total), again with balanced ratios (49.5–50.5%) between up- and downregulated genes (Supplementary Table S7). 

Similar to what was observed for GATA4, the majority (80%) of GATA6 DEGs refer to protein-coding genes. However, beyond these ratios we did observe stronger effects in terms of differential expression change for the DE lncRNAs compared to DE protein-coding genes. More specifically, the average fold change of lncRNAs in the GATA4 dataset was ±0.92 compared to ±0.63 for the protein-coding genes. This pattern was confirmed also in the GATA6 dataset in which we observe average fold changes of 0.87 and 0.66 for lncRNA and protein-coding genes respectively. In sum, our RNA-seq data suggest extensive yet balanced changes in the transcriptome of GC cells following KD of either GATA4 or GATA6, which can be summarized to an increased number of affected protein-coding genes accompanied by stronger changes in expression for the affected lncRNAs.

**Figure 3:**
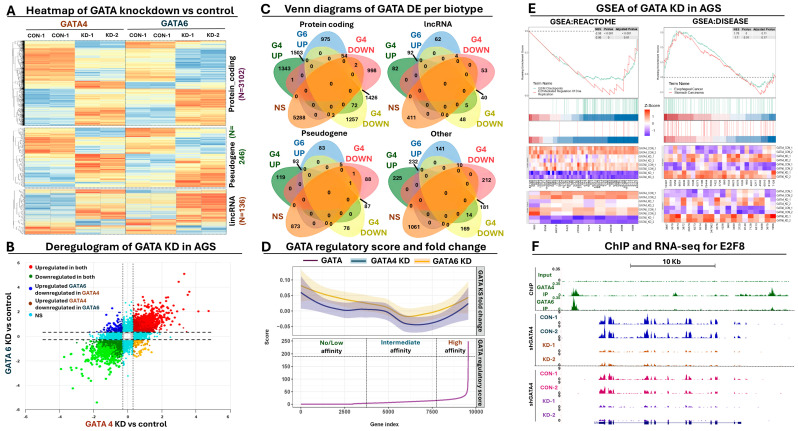
Transcriptome analysis following inducible GATA RNAi in AGS cells. (**A**) Heatmap summarizing the normalized expression (z-score) of the commonly affected differentially expressed genes (DEGs) across all samples. (**B**) Deregulogram depicting the correlation of expression in GATA4 KD (x-axis) vs GATA6 KD (y-axis). Colors represent the level of statistical significance across both datasets. (**C**) Venn diagrams summarizing the number of commonly affected DEGs between both datasets across coding and non-coding biotypes. (**D**) Deregulogram highlighting the association between GATA presence at gene loci with the deregulation of the corresponding transcripts. Bottom plot ranks all genes according to their GATA regulatory score, upper plot highlights the smoothed pattern of deregulation (fold change) of the same sorted genes for GATA4 (shown with blue) and GATA6 KD (shown with yellow). (**E**) GSEA analysis summarizing the enrichment of genes involved in cell cycle regulation (left) and upper gastrointestinal cancer (right). Heatmaps at the bottom highlight the expression of each enriched gene (indicated via entrez ID at the bottom) across all RNA-set data from both datasets, shown at the right of each heatmap. (**F**) UCSC browser snap-shot highlighting the E2F8 locus as an example of a GATA-deregulated target. Green tracks summarize GATA ChIP-seq peak, blue/orange tracks summarize control and KD RNA-seq expression for GATA4, while pink/purple tracks summarize control and KD RNA-seq expression for GATA6.

Next, we directly compared the two GATA-affected transcriptomes to identify common DEGs following GATA4/6 KD. This analysis revealed 3.884 commonly affected genes, corresponding to ~30% of all DEGs between both datasets. Expression of the remaining genes was significantly affected in one of the two GATA KD datasets (Supplementary Figure S3A). As expected, most of these common DEGs referred to protein-coding targets, while the non-coding DEGs mainly consisted of pseudogenes and lincRNAs (Figure 3A). Importantly, expression of these common DEGs was unidirectionally affected between the two GATA KD datasets, aligning them across the diagonal when a deregulogram analysis was performed (Figure 3B). Separate comparison of the up- and downregulated DEGs across different biotype categories revealed 1.502 and 1.426 commonly up- and downregulated protein-coding DEGs between the two datasets, in contrast to 417 and 308 commonly up- and downregulated non-coding RNAs, respectively (Figure 3C). To summarize, our transcriptome analysis revealed the existence of unidirectionally affected coding and non-coding GATA4/6 target genes, although each transcription factor retained a pool of uniquely regulated coding and non-coding targets in GC cells. 

#### 3.3.2. Calculation of GATA Regulatory Score and Correlation with Differential Expression

To gain additional insights regarding the direct involvement of GATA4 and/or 6 in the observed transcriptional changes, we took advantage of the ChIP- and RNA-seq data in AGS cells and performed a GATA regulatory score calculation. This algorithm considers parameters such as relative enrichment, number, and proximity of ChIP peaks over a gene locus to calculate a score that summarizes the involvement of a transcription factor in the regulation of the corresponding transcript. Once calculated, the regulatory score can be used as an index for sorting DEGs, facilitating associations of chromatin interactions with transcriptional changes from RNA-seq data. 

Based on the regulatory score analysis, we observed small transcriptional fluctuations as a general trend for genes with zero or low regulatory scores (Figure 3D). This trend was consistent for both transcriptomes (GATA4 and 6 KD), although in the GATA4 dataset, we did observe a more constant decline in expression compared to the GATA6 genes, for which the initial decline flattened out eventually. This pattern could be explained by a more or less equal mix of up- and downregulated transcripts that represent indirect targets of GATA4/6. At more intermediate regulatory scores, we observed a sharp decline as a general expression trend for both transcriptomes, suggesting that downregulation is more frequent for genes with few or weak GATA4 or 6 peaks under the conditions of our knockdown experiments. Interestingly, as the regulatory score gradually increased towards very high values, the deregulation effect was rapidly shifted towards a consistent upregulation, indicating that stronger GATA presence at the gene loci is more frequently associated with activation of gene expression. It should be noted, however, that these observations represent general trends and do not exclude the existence of up- or downregulated genes across the whole range of regulatory score calculations. Collectively, this analysis further supports the importance of GATA-mediated regulation in GC cells, serving as a criterium for selecting direct targets of these transcription factors for downstream analysis. 

#### 3.3.3. Functional Analysis of Differentially Expressed Genes

We subsequently subjected our differentially expressed genes to functional enrichment analysis, assessing their functional implications. GSEA analysis provided several lines of evidence for the deregulation of genes involved in cell cycle control when GATA4 or GATA6 function was impaired in GC cells (Figure 3E, Supplementary Table S8). The observed cell cycle effects included genes which were co-regulated by E2F factors and/or involved in G2/M checkpoint control, an observation that aligns well with the results of our ChIP-seq peak motif enrichment and functional analysis. Expression of such genes was generally reduced according to our RNA-seq results, suggesting that in the presence of both GATA factors, these genes are positively regulated (Figure 3F). Apart from the observed cell cycle effects, we also noticed enrichment of affected targets in the regulation of apoptosis and *TP53* activity in DNA repair, again in alignment with the ChIP-seq results and the observed cell cycle defects. Importantly, we also observed upregulation of *NURP1* upon KD of GATA. NURP1 has been shown to alleviate ROS generation, oxidative stress, and lipid peroxidation through inhibition of ferroptosis, a special form of programmed cell death that consumes antioxidant enzymes, promoting lipid peroxidation and oxidative stress. Other functionally enriched categories included keratinocyte development, cholesterol metabolism, epithelial morphogenesis, DNA replication, and various forms of DNA repair, suggesting that impairment of GATA function drives transcriptional changes that radically affect the homeostasis of GC cells (Supplementary Table S8). 

Through RT-qPCR experiments, we confirmed the deregulated expression of several genes involved in the observed functional categories in response to GATA4 or 6 KD in AGS cells (Supplementary Figure S3B, Table S9). Importantly, none of these target genes significantly altered its expression in response to scrambled shRNA expression in AGS cells (Supplementary Table S9). To gain extra confidence, we moved beyond AGS and independently checked with RT-qPCR the expression levels of the same target genes in MKN45 GC cells, in which GATA4 and 6 are also expressed in lower yet detectable levels compared to AGS. Both transcription factors were silenced using lentiviral-mediated shRNAs transductions (Supplementary Figure S2C,D, Table S6). In all cases, we observed a very similar pattern of deregulated target gene expression following KD of GATA4 or 6 in MKN45 (Supplementary Figure S3C, Table S9) compared to the AGS results, which collectively confirms our RNA-seq data along with the regulatory function of both GATA factors on these target genes. 

In addition, we complemented the GSEA results with classical gene ontology analysis for the deregulated GATA targets, which not only confirmed the GSEA and RT-qPCR observations but further enriched them with the involvement of genes in the metabolic process of reactive oxygen species along with cytochrome P450-mediated metabolism of xenobiotics (Supplementary Figure S3D, Table S10). This analysis also provided insights regarding the deregulation of genes involved in wound healing responses, vasculature development, and angiogenesis, which not only points towards the expected phenotypes of GATA4/6 KD in GC cells (see below) but also confirms the involvement of GATA-mediated functions in GC progression and metastasis beyond initial carcinogenesis (Figure 1, Supplementary Table S10). In terms of disease involvement, we detected upregulation of genes associated with esophageal and stomach cancer, again supporting the role of these transcription factors as master regulators of neoplastic development in the upper GI tract (Figure 3E). Other disease categories that were enriched included colitis, GI system, and inflammatory bowel disease (Supplementary Table S10). Moving beyond GI cancer, we also observed enrichment of genes involved in breast and lung cancer, melanoma, teratoma, and central nervous system malignancies, again in alignment with the results from the ChIP-seq peak functional enrichment (Figure 2E). 

Taken together, functional enrichment analysis of RNA-seq data aligned well with the results from the GATA ChIP-seq analysis, providing several independent lines of evidence according to which GATA function drives GC through the regulation of genes involved in organ development, regulation of cell cycle and apoptosis, and DNA repair. In addition, these data unveil a new molecular role for GATA-mediated regulation in the response to oxidative stress in GC cells, underlying the multi-faceted role of GATA lineage survival function in cellular homeostasis and disease. 

### 3.4. In Vitro Phenotypic Characterization of GATA4 and GATA6 Knockdown

Having established a role for GATA4 and 6 chromatin interactions in transcriptional changes that affect cancer-related processes in GC cells, we subsequently utilized our shRNA strategies to dissect the phenotypic impact of both transcription factors both in AGS and MKN45 GC cells. This approach allowed us to challenge our GATA4/6 RNA-seq and RT-qPCR conclusions through conduction of colony formation and wound healing assays, flow cytometric analyses of the cell cycle profile and apoptosis, as well as evaluation of oxidative stress responses.

#### 3.4.1. Colony Formation and Wound Healing Assay

**Figure 4:**
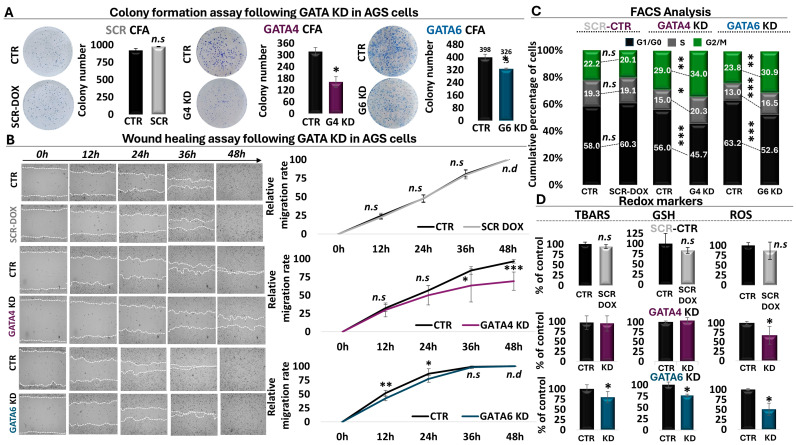
Phenotypic analysis of GATA KD in gastric cancer cells. (**A**) Colony formation assay following inducible knockdown of GATA4 and 6 in AGS cells. An inducible scrambled shRNA is also shown as a control. (**B**) Wound healing assay across a two-day time course of GATA4 and 6 knockdown in AGS cells vs the scrambled control. Magnification is 10x. (**C**) Cell cycle profiling of GATA4 and 6 downregulation in AGS cells align with the scrambled control. Black bars represent G1/G0, grey bars represent S phase, and green bars represent G2/M phase. (**D**) Analysis of anti-oxidation effects associated with scrambled shRNA expression or shRNA-mediated impairment of GATA4 and 6 function in AGS cells. * *p*-value ≤ 0.05, ** *p*-value ≤ 0.01, *** *p*-value ≤ 0.001, n.s: not significant, n.d: not detectable.

Silencing of GATA4 in AGS had a significant impact on cell proliferation capacity. More specifically, in our colony formation assays, we observed a statistically significant 50% decrease in the number of colonies formed following GATA4 KD, along with a milder, yet again statistically significant, decrease of 20% in colonies formed in the case of GATA6 KD (Figure 4A). We also observed statistically smaller colonies following KD of GATA4, in contrast to GATA6, in which we did not detect any significant effects in colony area (Figure 4A, Supplementary Table S11). Of note, we did not detect any significant effects in AGS cells expressing the scrambled shRNA control. Importantly, we observed similar reduction in the ability of MKN45 cells to form colonies following knockdown of GATA4 (associated with a 57% decrease in colony number) or GATA6 (associated with a 69% decrease in colony number) compared to the scrambled control (Supplementary Figure S4A, Table S11). 

To further investigate the role of both factors in cancer-related processes, a wound healing assay was conducted. In the case of GATA4 KD, this assay revealed that the migration rate of AGS cells began to decline 24 h post-doxycycline administration and became significantly reduced at 36 h, while the effect became even more pronounced at 48 h post-doxycycline addition (Figure 4B, Supplementary Table S11). These findings were corroborated by the wound healing assay of GATA6 KD, which displayed a similar trend of reduced AGS cell migration capacity at 12 h and 24 h post-knockdown induction (Figure 4B, Supplementary Table S11). Interestingly, although the effect of GATA6 on cell proliferation was less pronounced than that of GATA4, it appeared to precede the latter and to diminish once the GATA4 effect initiated, possibly providing hints regarding the temporal involvement of these factors in the process of wound healing. Again, we did not observe any effects in the scrambled control in AGS cells (Figure 4B, Supplementary Table S11). Importantly, we independently confirmed these observations in MKN45 cells, in which, again, the GATA6 effect not only preceded GATA4 but was stronger (Supplementary Figure S4B, Table S11). Collectively, these data fully align with the results from the ChIP-seq, RNA-seq, and RT-qPCR analyses (Figure 2 and Figure 3), emphasizing the oncogenic role of GATA4 and 6 both in AGS and MKN45 GC cells in agreement with the clinical observations (Figure 1).

#### 3.4.2. Flow Cytometry for Cell Cycle Profiling and Analysis of Apoptosis

To delineate in detail the mechanism by which GATA4/6 promote cell proliferation, we analyzed the cell cycle profiles of GATA4 and GATA6 knockdown by flow cytometry in AGS and MKN45 cells. As shown in Figure 4C, cell cycle profiling revealed a similar pattern after shRNA-mediated KD of both transcription factors in gastric cells. More specifically, in the absence of GATAs, AGS cells exhibited a decrease in the G0/G1 phase, indicating fast entry into the cell cycle. Subsequently, there was a notable accumulation of cells in the S phase and even more in the G2/M phase following GATA4 or 6 KD (Figure 4C, Supplementary Figure S5A, Table S12). Specifically, in the GATA4 knockdown condition, we observed a 10.3% decrease in the G0/G1 phase, a 5.3% increase in the S phase, and a 5% increase in the G2/M phase. The GATA6 knockdown condition was associated with a 10.6% decrease in the G0/G1 phase, a 3.5% increase in the S phase, and a 7.1% increase in the G2/M phase, overall suggesting that AGS cells are arrested in the S and G2/M phases, as evidenced by the increase in the proportion of cells in the corresponding phases. Similar effects were also observed in MKN45 cells (Supplementary Figures S4C and S6A, Table S12). Therefore, the increase in the G2/M phase, combined with the results of the colony formation assay, suggest a dysregulation in the G2/M checkpoint leading to G2/M arrest. Particularly noteworthy is the temporal correlation of the cell cycle data with the rapid reduction that we observed in cell proliferation of GATA4 KD AGS cells during the time course of the wound healing assay, possibly suggesting that the observed wound healing decline could be attributed in part to the cell cycle arrest. 

To further investigate a potential link between the observed cell cycle dysregulation and programmed cell death, a FACS analysis for apoptosis was also conducted in both cell lines. The results showed a significant increase in the proportion of AGS cells in the late apoptotic state (Supplementary Figure S5B, Table S12) and MKN45 cells in both early and late apoptotic states (Supplementary Figure S6B, Table S12) following GATA4 KD in both cell lines. Knockdown of GATA6 exhibited a statistically significant increase both in early and late apoptotic states in both cell lines. Taken together, the data from the cell cycle profile and apoptotic analyses highlighted the regulatory role of GATA4 and GATA6 in the cell cycle process of GC cells, particularly in the regulation of the G2/M checkpoint, which is reflected in the observed induction of apoptosis and can be linked to wound-healing effects.

#### 3.4.3. Analysis of Oxidative Stress Responses

Several studies have revealed a role for oxidative stress responses in cancer-related processes, such as DNA damage and apoptosis. Having observed enrichment of oxidative stress responses and ferroptosis in our RNA-seq and RT-qPCR results, and given that to the best of our knowledge, nothing is known for GATA4- or 6-mediated effects in these processes in GC cells, we decided to utilize our KD strategies to challenge the role of both factors in redox regulation of GC cells. Our redox screen included three factors, namely reactive oxygen species (ROS) as a general indicator of oxidative stress, glutathione (GSH) as a general regulator of antioxidant defense, and thiobarbituric acid reactive substances (TBARS) as a marker of oxidative damage. The flow cytometric and spectrophotometric analysis of redox markers revealed changes in the redox profile of AGS cells with impaired GATA4 and 6 function (Figure 4D, Supplementary Table S13). More specifically, GATA4 KD resulted in a statistically significant decrease in ROS levels (32.6%) compared to the control group. However, our analysis did not reveal statistically significant effects on any of the other markers examined in AGS cells under the conditions of our experiment. However, we did observe a statistically significant reduction in TBAR levels following GATA4 KD in MKN45 cells (Supplementary Figure S4D, Table S13). Conversely, in AGS cells, KD of GATA6 was associated with a statistically significant decrease in GSH (23.5%), ROS (49.6%), and TBARS (20.4%) levels compared the control condition or the scrambled shRNA (Figure 4D, Supplementary Table S13). It should be noted, however, that we did not observe any statistically significant effects for GATA6 KD in MKN45 cells (Supplementary Figure S4D, Table S13). Taken together, these observations suggest that in AGS cells, KD of either GATA4 or GATA6 is associated with a decrease in ROS levels for the cell. However, involvement of GATA6 seems to be more pronounced, allowing the cell to consume GSH in order to decrease ROS levels. As a result, cells can contain oxidative damage as shown through the observed decrease in TBAR levels. 

### 3.5. Characterization of GATA-Regulated Molecular Signatures in Cancer Patients

Given the involvement of GATA-mediated targets in various cancer-related processes, we sought to explore the possibility of isolating molecular signatures consisting of coding and non-coding GATA targets with diagnostic and prognostic potential for GC. For this analysis, we took advantage of our GATA regulatory score calculation along with our access to and analysis of a multi-cancer TCGA panel, challenging again the clinical significance of our findings in cancer biopsies. 

#### 3.5.1. Establishment of Metagene Signatures from Coding and Non-Coding GATA Targets

The selection criteria for composing our metagene signatures included the presence of GATA peaks at the vicinity of their loci along with deregulation in the RNA-seq data. More specifically, we selected coding and non-coding genes for which (i) the GATA regulatory score was ≥10, a threshold that matched the regulatory score median of our analysis, facilitating isolation of targets with strong GATA presence at the vicinity of their loci; (ii) their expression was equally and unidirectionally affected by GATA4 and 6 KD, suggesting their common GATA-mediated regulation; and (iii) the fold change of their expression was at least equal to or higher than ±1.25 for both transcription factors and of course fulfilled the criterium of statistical significance. This combined filtering facilitated the isolation of 104 coding and non-coding GATA targets (Supplementary Table S14). 

**Figure 5:**
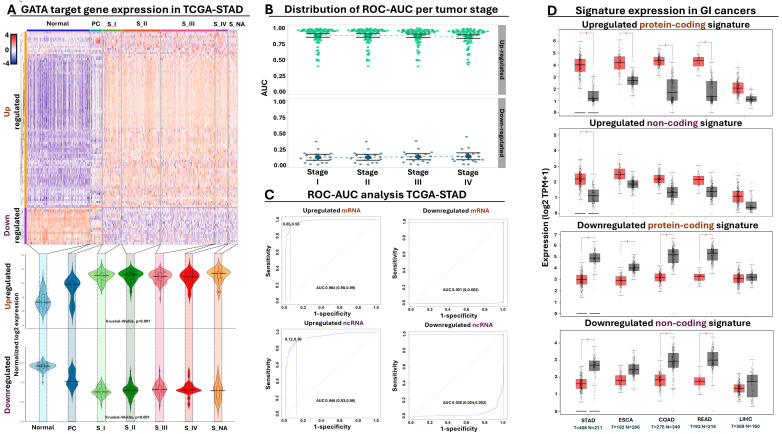
GATA meta-signature clinomic footprint in cancer biopsies. (**A**) Heatmap summarizing the normalized expression (z-score) of the commonly affected differentially expressed genes (DEGs) across all TCGA STAD samples. Violin plots at the bottom summarize the expression of the up- and downregulated DEGs across all tumor stages. Statistical significance refers to normal vs rest and was calculated with the Kruskal–Wallis test. (**B**) Beeswarm plot summarizing the distribution of ROC AUC across all gastric tumor stages for all 104 DEGs from (**A**), divided according to their expression (up- or downregulated). (**C**) ROC curve analysis highlighting the diagnostic power of the four selected meta-signatures. Average AUC performance is indicated along with 95% confidence intervals and sensitivity/specificity values. (**D**) Boxplots comparing the expression of the four selected meta-signatures in normal (grey) vs tumor (red) biopsies across various gastrointestinal tumors. * *p*-value ≤ 0.05.

The majority (86 out of 104, corresponding to 83%) of our selected genes were upregulated in GC biopsies, confirming the oncogenic function of GATA-mediated regulation in gastric biopsies beyond the cell line level (Figure 5A). Importantly, the observed up- or downregulated pattern of our metagene panel was statistically significant both in early and advanced stages of gastric tumors compared to normal or paracancerous expression, pointing towards the potential of GATA-mediated targets for early GC detection and tumor staging (Figure 5A). 

#### 3.5.2. Evaluation of Diagnostic and Prognostic Potential in GC Patients

Differential expression is considered a prerequisite for tumor detection but is not always indicative of a strong diagnostic potential. Therefore, we subjected our metagene panel of 104 genes to ROC AUC analysis, designed to calculate the diagnostic power of each gene for gastric tumor detection. We independently performed this analysis across all tumor stages, ensuring isolation of metagene signatures with elevated diagnostic potential from early stages of gastric tumor development (Supplementary Table S15). Indeed, this analysis confirmed that the vast majority of our selected GATA targets held great promise as diagnostic markers for tumor presence, with an AUC median of 0.93 for tumor detection in the upregulated genes and 0.11 (corresponding to 0.89 for normal epithelium detection) for the downregulating genes across all stages (Figure 5B). 

Encouraged by the elevated diagnostic potential of our selected targets, we isolated four compact GATA-regulated meta-signatures composed of (i) the upregulated protein-coding genes *MYB*, *KIF11*, *CKS2*, *CASC5*, and *SMC2*; (ii) the upregulated non-coding genes *RP11-424C20.2*, *PVT1*, and *RP11-160E2.6*; (iii) the downregulated protein-coding genes *KIAA1875*, *GADD45B*, *FAM189A2*, *CKB*, and *IL11RA*; and finally, (iv) the downregulated non-coding genes *CTC-510F12.2*, *SLC25A21-AS1*, and *NSUN5P1*. The elevated diagnostic power of each meta-signature was confirmed again in gastric tumors, supporting their diagnostic potency for early tumor detection (Figure 5C). Moving beyond diagnosis, we detected statistically significant changes in the combined expression of our meta-signatures across gastric tumors stratified according to their MSI status or Lauren classification criteria (Supplementary Figure S7A). We also subjected our tumor-expressed meta-signatures to Kaplan–Meier analysis, detecting a statistically significant shorting of overall survival time along with a statistically significant probability for lymph node infiltration when meta-signature expression was elevated in gastric tumors (Supplementary Figure S7B). Collectively, these observations underline the importance of GATA-mediated regulation for isolating diagnostic markers suitable for tumor detection and prognosis, irrespective of their histological or molecular classification. 

#### 3.5.3. GATA Meta-Signature Expression in a Multi-Cancer Panel

Having established the diagnostic and prognostic potency of our GATA-regulated meta-signatures, we expanded the analysis of their expression to other types of cancer. We initially focused on GI tumors, comparing the expression of all four meta-signatures in gastric, esophageal, liver, and colorectal tumors against their normal counterparts. With regards to the upregulated protein-coding meta-signature, we detected statistically significant over-expression in all GI tumors apart from the liver (Figure 5D). Interestingly, when we checked the expression of the upregulated non-coding meta-signature, statistically significant overexpression was detected only in gastric tumors but not in the remaining cancers of this GI panel. This observation could be attributed in part to a higher tendency for cancer-specific expression of the non-coding transcripts compared to their coding counterparts, although we certainly cannot exclude other possibilities, such as a lower level of expression that could introduce intra-tumoral and/or inter-patient variability, influencing the statistical significance of this meta-signature. 

Expression of the downregulated protein-coding meta-signature agreed well with the previous observations since we detected statistically significant downregulation in all GI tumor types compared to their normal counterparts, with the exception of liver neoplasms. The non-coding signature was again more selective in terms of differential expression since we did not detect statistical effects for esophageal or liver tumors, yet this time, downregulation of this meta-signature was significant in colorectal tumors. The importance of GATA-mediated regulation in multiple forms of cancer is further supported by the observation that several coding and non-coding members from our initial meta-signature panel of 104 GATA regulated targets (Supplementary Table S14) were not only differentially expressed in GI tumors but also in cancers with entirely different system origins (Supplementary Figure S7C). Taken together, these observations collectively highlight the deregulated expression of our selected GATA-regulated meta-signatures across a multi-cancer panel, providing tantalizing evidence for their potential diagnostic and prognostic application in GC and beyond.

## 4. Discussion

The master regulatory role of GATA4 and GATA6 in organogenesis and cell specification, as well as their lineage survival nature, challenges the investigation of their broad function in gene-regulatory molecular pathways in cancer. Our study offers functional insights regarding GATA4 in GC, revealing its oncogenic implications in cell cycle progression and apoptosis. GATA4’s molecular function has been primarily studied in cardiac hypertrophy [68,69,70], while in cancer, it has been shown to act as a tumor suppressor in pancreatic adenocarcinoma, lung, and colorectal cancers [71,72,73]. In pancreatic cancer, GATA4 knockdown increased the ability to form colonies in CFPAC-1 cells, while its in vivo overexpression inhibited tumor growth [73]. Similarly, evidence from in vitro and in vivo experiments in breast cancer revealed that GATA4 again functions as a tumor suppressor, demonstrating inhibitory effects on breast cancer cell proliferation and migration while promoting apoptosis [74]. Interestingly, a recent study conducted in a bile acid-induced gastric intestinal metaplasia cell model revealed that activation of NF-κB signaling promoted expression of GATA4 and CDX2, leading to the transactivation of MUC2 [75]. Additionally, they showed that GATA4 and CDX2 transactivated each other, revealing the complex and cooperative function of lineage-specific factors in the regulation of downstream targets. Consistent with our findings, other studies have also reported the oncogenic role of GATA4 in GC cell lines, focusing on the oncogenic transcriptional network that GATA4/GATA6/KLF5 cooperatively promote to drive GC tumorigenesis [37]. In addition, the *GATA4* and *6* loci have been shown to undergo genomic amplification in gastric tumors [76,77,78], which further highlights their oncogenic role in these tumors and aligns well with the observed upregulated levels of both factors in our patient sample analysis. Taken together, these reports suggest that GATA4 acts as a double-edged sword in carcinogenesis, with functions that depend on its cellular content.

Our investigation into the impact of GATA6 impairment on cell phenotype revealed a notable inhibition of apoptosis, resulting in an increased proportion of cells undergoing early and late apoptotic processes. This coincided with G2/M cell cycle arrest, aligning well with a previous study conducted on AGS, Caco2, and SNU16 GC cell lines [79]. The study intriguingly demonstrated that GATA6 associates with various protein partners, including GATA4, to indirectly regulate downstream targets, one of which is the CDX2 intestinal metaplasia marker. Conflicting to these findings, a different study suggested that lentiviral-mediated knockdown of GATA6 in MKN45 and AGS cells promotes migration and invasion by modulating miR-520b/CREB1. In that study, injection of MKN45 cells expressing shRNAs against GATA6 into nude mice led to an increased incidence of lung metastasis in vivo [80]. These conflicting findings could be attributed in part to different shRNA sequences that possibly affect other GATA members or could stem from the different methodologies of impairing GATA function, since our inducible RNAi system allowed direct comparison of control and KD effects in the same genetic background, in contrast to lentiviral experiments, in which control and KD effects can be confounded by differences in viral titer. In agreement with our findings, GATA6 exhibited an oncogenic function in colorectal cancer through repression of BMP expression, contributing to invasion and metastasis through regulation of the uPA protease [81,82]. Conversely, in cancer types unrelated to GI, like lung cancer, GATA6 has been shown to inhibit cell migration and proliferation of LSCC cells by downregulating Shh [83]. This dualistic nature of GATA6, serving either as an oncogene or a tumor suppressor, is evident even within the same cancer type, as seen in pancreatic ductal adenocarcinoma. In this context, GATA6 can exhibit an oncogenic function by influencing cell proliferation [84,85] while also acting as a tumor suppressor by inhibiting EMT transition in vitro, impeding cell propagation in vivo [86]. Collectively, these studies emphasize the intricate and multifaceted involvement of lineage survival factors such as GATA6 in tumor progression, highlighting their stage- and content-specific impact [87]. In addition, these observations also stress on the difficulty of utilizing these factors as diagnostic and therapeutic agents, despite their central roles in carcinogenesis. Through the integration of ChIP and RNA-seq data, our study provides, for the first time, thorough and integrated molecular evidence supporting that GATA4 and GATA6 collectively play an oncogenic role in GC by influencing both the cell cycle process and apoptosis.

At the chromatin level, master regulators such as GATA4 and GATA6 pose an additional challenge in pinpointing their precise molecular effects due to their involvement in intricate regulatory networks, which encompass feedback loops and interactions with other transcription factors [88,89]. The data presented above reveal a distinct distribution of GATA4 at the promoter region of genes, accompanied by epigenetic markers H_3_K_27_ac and H_3_K_4_me3, highlighting its robust binding at active regulatory sites. The DNA binding efficiency of GATA4 is influenced by the transcriptional co-activator/p300, which acetylates it. This acetylation, in turn, promotes GATA4 multimerization, enhancing its DNA-binding capacity notably in cardiomyocytes [90,91]. Furthermore, the co-occurrence of GATA4 and H_3_K_27_ac at the chromatin has been investigated in the context of cardiac hypertrophy, illustrating GATA4’s role in regulating H_3_K_27_ac deposition by recruiting p300/CBP to active regulatory regions, particularly enhancers [92]. GATA presence in enhancer regulatory regions aligns with our observations of GATA6 distribution in distal intergenic regions, marked by H_3_K_27_ac and H_3_K_4_me1, indicating occupancy of enhancer elements. Moreover, studies have indicated that GATA6, in conjunction with other pioneer factors, plays a crucial role in the remodeling of chromatin structure at downstream target sites in hiPSCs [51]. This fits well with the reported chromatin association of GATA6 with CTCF, warranting further investigation [93,94].

In our study, we utilized RT-qPCR to demonstrate the dysregulation of specific target genes identified through our RNA-seq analysis. Among the cell proliferation targets analyzed, *PDGFRA*, *E2F8*, and *EMP2* exhibited downregulation, while *ARHGEF2* and *DDIT4* showed upregulation in both AGS and MKN45 cells following suppression of GATA4 and GATA6. The PDGF/PDGFR signaling pathway is a commonly deregulated pathway in various cancers [95,96] often associated with drug resistance, leading to the development of several targeted therapeutic strategies [97]. For instance, PDGFRA (Platelet-Derived Growth Factor Receptor Alpha) has been implicated in the promotion of cell proliferation in peripheral T-cell lymphoma [98] and is negatively regulated in gliomas through an ERK-dependent mechanism that reduces cell proliferation [99]. In GI stromal tumors (GIST), PDGFR mutations influence the development of personalized therapeutic strategies [100]. 

The connection between cell cycle and transcription factors belonging to the E2F family is well established [101]. Bioinformatic transcriptome analysis of GC biopsies revealed that E2F8 is highly expressed in tumors, affecting the overall survival of patients [102]. E2F8, a multifaceted transcription factor, exhibits both oncogenic and tumor suppressive activities in cancer, depending on the specific tumor type [103,104]. Cyclin-dependent kinase (CDK) 4/6-cyclin D complexes, which act as a cell cycle checkpoint regulator, phosphorylate the retinoblastoma (Rb) tumor-suppressor protein and release E2F1 from Rb, which triggers the transcription of G1-S transition and S phase genes [105]. This process controls the transcriptional activity of E2Fs [106]. The strong effect of the (RB)-E2F pathway in GC progression [107], along with the cancer-promoting effect of E2F8 [108], agrees with our results, since the observed inhibition of cell cycle progression could be explained in part through the noted downregulation of E2F8 upon GATA KD. 

A different GATA target gene in our study, EMP2 (Epithelial membrane protein 2), is poorly studied in GC. It is known that EMP members can be implicated in cell migration and metastasis processes [109]. Specifically, EMP2 was recently shown to be implicated in G2/M cell cycle arrest through regulation of the G2/M checkpoint in vitro in bladder cancer [110,111], an observation that aligns well with the downregulation of EMP2 along with the G2/M arrest effects in our study. ARHGEF2 was also shown to be affected by GATA KD. Recent, in vivo evidence on GC showed that IL-6/STAT3 signaling can be activated in the tumor environment through persistent inflammation. In parallel, STAT3 was shown to directly block transcription of *miR-520f-3p*. Notably, one of the targets of *miR-520f-3p*, NEK9, is implicated in the phosphorylation of ARHGEF2, which, in turn, activates RhoA, promoting enhanced cell motility and facilitating both local and distant cancer cell spread [112]. Research in diffuse large B-cell lymphoma highlights the direct regulation of ARHGEF2 by STAT3 in enhancement of cell migration [113]. Mirroring observations come from pancreatic cancer studies, according to which ARHGEF2 (also known as GEF-H1) is implicated in promoting cell growth and invasiveness [114]. Similarly, DDIT4 has been identified as an oncogene in GC, influencing cell cycle progression and apoptosis through the p53 and MAPK signaling pathways [115]. Our research reveals that ARHGEF2 and DDIT4 are upregulated when GATA4 and GATA6 are impaired. Their combined function on cell proliferation could explain in part the observed release from the G1/G0 phase upon GATA KD, leading to G2/M arrest through the impairment of independent factors such as EMP2 discussed above. However, we cannot exclude the possibility that the observed upregulation is the result of a transcriptional response of cells experiencing the tumor-suppressive effects of GATA knockdown, since both target genes generally exhibit oncogenic properties in GC.

In terms of cell migration, our analysis revealed upregulation of the target genes *UNC5B*, *PHLDA1*, and *KRT6A*. UNC5B has been shown to induce G2/M cell cycle arrest in bladder cancer, inhibiting tumor growth both in vitro and in vivo through the dephosphorylation of P53 [116]. A separate study on the same cancer highlighted UNC5B’s role in cell migration, invasion, and metastasis by interacting with key migration markers such as fibronectin, beta-catenin, and vimentin [117]. Ectopic overexpression of PHLDA1 has been shown to effectively reduce cell proliferation, migration, and invasiveness in GC cells. Furthermore, KRT6A is recognized for its involvement in cell migration and the epithelial–mesenchymal transition (EMT) process across various cancer types, such as lung adenocarcinoma [118,119], colorectal [120], and pancreatic cancer [121]. Although research on KRTGA in GC is limited, studies have indicated that KRTGA is upregulated as a downstream target of MAPK1 upon impairment of the latter in AGS cancer cells [122]. The observed upregulation of KRTGA is associated with effects on cell migration and invasion, aligning with our findings on the impact of GATA’s impairment on KRTGA expression. Taken together, these validated effects of GATA targets genes in both GC cell lines, along with the functional analysis of the integrated ChIP and RNA-seq results, provide mechanistic support for the observed cancer-related effects that accompany GATA KD under our experimental conditions. 

Cancer cells are constantly exposed to a strong oxidizing environment characterized by increased ROS generation as a consequence of their high metabolic rate [123]. In order maintain redox equilibrium and counterbalance the detrimental effects of oxidative stress under these adverse conditions, cancer cells have developed a complex regulation of the antioxidant machinery [124]. Therefore, it has been proposed that targeting factors which control oxidative stress in cancer cells could be a promising therapeutic strategy [125]. To address this hypothesis, we relied on the analysis of three reporter assays, examining the effects of impaired GATA4 and 6 function on the redox phenotype of AGS GC cells. More specifically, we measured GSH, which is the most prevalent and significant cellular non-protein thiol, acting as an antioxidant either directly by scavenging reactive species or indirectly as a substrate for various antioxidant and detoxification enzymes [126]. We also measured ROS, which act as extremely reactive chemical moieties that at low levels regulate several physiological functions, such as cell signaling, defense against pathogens, and gene expression, whereas at high levels cause severe oxidative damage to macromolecules [127]. In addition, we also analyzed TBARS, which are chemical adducts produced by the reaction between MDA and TBA and are considered reliable indicators of lipid peroxidation [128]. 

According to our phenotypic analysis, KD of GATA4 and 6 induced redox adaptations in AGS GC cells. In particular, disruption of GATA4 activity led only to the significant reduction of intracellular ROS levels. However, disruption of GATA6 activity had a broader impact on redox state, significantly lowering GSH, ROS, and TBARS levels. It is well-established that GSH contributes to the neutralization of excessive ROS levels either as a direct free radical scavenger or as an electron donor used by glutathione peroxidases. The latter convert GSH to its oxidized form, glutathione disulfide (GSSG), which can be regenerated back to its active, reduced form by glutathione reductase [129]. Hence, the observed depletion of the intracellular GSH pools upon GATA6 KD could be attributed to its consumption for eliminating the elevated ROS levels, providing cellular protection against oxidative damage. This hypothesis is further supported by the observed depletion of TBARS, suggesting inhibition of lipid peroxidation. These phenotypic findings manifest redox adjustments, collectively supporting the notion that downregulation of GATA6 in cancer cells provides resistance to oxidative stress through redox regulation [130].

Our study also provides a possible mechanistic link for the aforementioned phenotypic effects of GATA impairment on redox balance. According to the results of the RNA-seq analysis and the qPCR validations, KD of both GATA4 and 6 induced the expression of the nuclear protein-1 (NUPR1) both in AGS and MKN45 cells. NUPR1, a highly expressed protein in cancer cells, is activated in response to stress signals, including oxidative stress, playing a pivotal role in regulating redox processes [131]. More specifically, NUPR1 is activated in the presence of excessive ROS levels to prevent cell death, predominantly via inhibition of ferroptosis [132]. Ferroptotic cells exhibit low GSH levels and glutathione peroxidase 4 activity, causing ROS accumulation in the cellular environment [133]. In our conditions, GATA-mediated upregulation of NURP1 coincided with a reduction in ROS and TBARs in GATA6, suggesting that NURP1 could be in part responsible for the observed antioxidizing effect of GATA impairment, possibly through inhibition of ferroptosis. 

Previous reports investigated the relationship between GATA family members and oxidative stress responses in cancer cells. For example, two isoforms of GATA1, GATA1-_FL_ and GATA-1_S_, exert distinct effects on redox homeostasis in K562 human myeloid leukemia cells. To be more specific, cells overexpressing GATA1-_FL_ show elevated cytosolic ROS levels and decreased GSH levels, experiencing severe oxidative stress that can lead to apoptotic cell death. In contrast, cells overexpressing the GATA-1_S_ isoform exhibit high mitochondrial ROS levels along with increased GSH levels and SOD1 activity, hence promoting leukemogenesis [134]. With regards to GATA4, its upregulation in colorectal cancer cells adapted to acidosis activates the transcription factor NF-kB, an important regulator of oxidative stress responses that elevates cytokine expression, decreasing ROS levels and suppressing apoptosis [135]. Ectopic expression of GATA4 in hepatocellular carcinoma cells activates NF-kB, resulting in cellular senescence [136]. With regards to GATA6, its miRNA-mediated knockdown in SW1990 pancreatic cancer cells inhibits cell proliferation, enhances ROS generation, and promotes cell death via apoptosis [85]. To the best of our knowledge, the results presented above connect for the first time the function of GATA4 and 6 to the regulation of redox state in GC cells, providing mechanistic insights at the molecular level through the regulation of NURP1. 

Beyond the observed effects on coding target genes, our results offer compelling evidence regarding the regulation of long non-coding RNAs by GATA4 and GATA6 in GC. The exploration of lncRNA regulation by lineage-survival transcription factors in GC is a promising and unexplored area of research. Our analysis provides direct insights regarding the identification of cancer-specific lncRNA targets for GATA4/6 that are suitable for specialized diagnosis and prognosis of GC. For example, we have shown that impairment of both factors upregulates the expression of LINC01003 and MALAT1 while downregulating DANCR and TPM3P9 in AGS cells. Previous studies have demonstrated that upregulation of LINC01003 plays a role in regulating cell migration through the CAV1/FAK signaling pathway in glioma [104]. However, the impact of GATA transcription factors on LINC01003 regulation has not been explored until now in any cancer type. On the other hand, MALAT1 was shown to promote oncogenesis of GC by inhibiting autophagic flux and inducing fibroblast activation [137]. Interestingly, in processes like osteogenic differentiation, MALAT1 expression is influenced by GATA4 [138]. Our findings establish, for the first time, a connection between the regulation of MALAT1 by GATA4 and GATA6 in GC, providing avenues for further research. Moreover, from the standpoint of the downregulated ncRNAs, the functional role of the TPM3P9 pseudogene in cancer remains unexplored. Intriguingly, the DANCR lncRNA is activated by the KLF5 master regulator in GC, known to undergo genomic amplification in this form of cancer [139]. As previously reported, KLF5 cooperates with GATA4 and 6, forming cooperative transcriptional networks that regulate downstream targets in a cancer- and tissue-specific manner [37]. Taken together, these results, along with the conclusion of our multi-cancer clinomic approach, provide a tantalizing list of lncRNA targets that are regulated by GATA4 and 6, exhibit cancer-specific patterns of expression even among related GI cancers, and constitute metagene signatures with elevated diagnostic and prognostic potency for GC. 

## 5. Conclusions

This study couples inducible RNAi-targeting of GATA4 and 6 with an integrated analysis of ChIP and RNA-seq data to molecularly dissect the cancer-related role of these lineage survival factors in GC cells. Through the combined experimental and computational strategy presented above, we provide mechanistic insights that link GATA-mediated regulation of coding and non-coding genes to cell proliferation and apoptosis, regulation of the redox state, and cell migration. In addition, this work provides a collection of four metagene signatures, consisting of coding and non-coding GATA4/6 targets, with elevated diagnostic and prognostic potency for GI cancer based on analysis in a multi-cancer panel. We propose this integrated analysis as a strategy for isolating and harnessing target genes from other lineage survival factors for diagnostic and prognostic applications based on their cancer-specific properties. Our current research focuses on this direction with the aim of expanding the available diagnostic and prognostic toolbox for GC in the near future.

## Figures and Tables

**Table 1:** Short hairpin RNA sequences.

Vector	Targets	Sense shRNA Sequence (siRNA Shown in Red)
pTER	Scrambled	GATCCCGTACAGCCGCCTCAATTCTTTCAAGAGAAGAATTGAGGCGGCTGTACTTTTTGGAAA
	GATA4	GATCTCGGACATAATCACTGCGTAATTCAAGAGATTACGCAGTGATTATGTCCTTTTTGGAAA
	GATA6	GATCTCGGTGATGACTGGTGCGGGATTCAAGAGATCCCGCACCAGTCATCACCTTTTTGGAAA
pSicoR	Scrambled	TGTACAGCCGCCTCAATTCTTTCAAGAGAAGAATTGAGGCGGCTGTACTTTTTTC
	GATA4	TGGACATAATCACTGCGTAATTCAAGAGATTACGCAGTGATTATGTCCTTTTTTC
	GATA6	TGGTGATGACTGGTGCGGGATTCAAGAGATCCCGCACCAGTCATCACCTTTTTTC

**Table 2:** Primer sequences used for RT-qPCR.

Primers	Primer Sequence
GATA4_Reverse	CCTCGGTGCTAGAAACACAA
GATA4_Forward	CCTGTGAGTTGGAGACTTCTTT
GATA6_Reverse	ACTTCAGATCAGCCACACAATA
GATA6_Forward	GTCGGTTCATGAGGTCTCTTATC
DANCR_Forward	CTGCATTCCTGAACCGTTATCT
DANCR_Reverse	GGGTGTAATCCACGTTTCTCAT
TPM3P9_Forward	TGCTGATGAGAGTGAGAGAGA
TPM3P9_Reverse	GCTTAGCTTCTTTGAGTTGGATTT
PDGFRA_Forward	CTGACAGTGGCTACATCATTCC
PDGFRA_Reverse	GAGCTGTGTCTGTTCCTCTTG
E2F8_Forward	AAGCCAACCAGCTCATCC
E2F8_Reverse	GACATCCTCTGTTGAGACTTCC
EMP2_Forward	AGCTACGGCTACTCCTACAT
EMP2_Reverse	TATTTGCGCTTCCTCAGTATCA
NUPR1_Forward	GACACTACACCCAGCAATAGAG
NUPR1_Reverse	GACTCAGTCAGCGGGAATAAG
LINC01003_Forward	GGTCGTCCTGCACTTTCTC
LINC01003_Reverse	GCTGCCTTCTGAGTCTTTGA
MALAT1_Forward	CATGACGGAGGTTGAGATGAAG
MALAT1_Reverse	AGCATTGCCCTTCTATTGGTATTA
ARHGEF2_Forward	AGTGGAACTGCTCTTGACAC
ARHGEF2_Reverse	ATTGAAGGTTCTGGCCTCAC
DDIT4_Forward	CCAAGACAGAGACGACTGAAC
1DDIT4_Reverse	AGCTTCCTGGGAAACACTATTC
UNC5B_Forward	AGTGAATGTGCCTGTGTGT
UNC5B_Reverse	TCTCTGTTCAGTCTCTCTCTCC
PHLDA1_Forward	ATCACGATGCAGGAAACGA
PHLDA1_Reverse	CAGTACATCATCGCTCCTAGAAA
KRT6A_Forward	GTTGGAGGTGGCTTCAGTT
KRT6A_Reverse	AGGAGGTGGTGGTGTACTT

## Data Availability

ChIP-seq FASTQ files are available with GEO accession numbers PRJNA224585 and GSE85467. RNA-seq FASTQ files are submitted to GEO under the accession number GSE274529. Access to controlled data is available with dbGAP (access approval TCGA #117269-1) and DUOS (access approval gTEX DUOS-DAR #350).

## Supplementary Materials

The following supporting information can be downloaded at: https://www.mdpi.com/article/10.3390/antiox13101267/s1. Supplementary Figure S1: ChIP-seq peak distribution, association and functional analysis in AGS cells. Supplementary Figure S2: Evaluation of knock-down efficiency for GATA4 and GATA6 in AGS and MKN45 cells. Supplementary Figure S3: RNA-seq data analysis of GATA KD in gastric cancer cells. Supplementary Figure S4: Phenotypic analysis of GATA4 and 6 KD in MKN45 cells. Supplementary Figure S5: FACS analysis of GATA KD effects on cell cycle and apoptosis in AGS cells. Supplementary Figure S6: FACS analysis of GATA KD effects on cell cycle and apoptosis in MKN45 cells. Supplementary Figure S7: Prognostic potential of meta-signature expression. Table S1: Statistical analysis of GATA4 and GATA6 transcription in TCGA cancer datasets. Table S2: Statistical analysis of GATA4 and GATA6 transcription in staged gastroesophageal tumors compared to normal biopsies. Table S3: Distribution percentage of all ChIP-seq peaks compared to TSS. Table S4: Sea motif enrichment analysis of GATA4 and 6 peaks. Table S5: Functional analysis of GATA ChIP-seq peak annotations. Table S6: Statistical analysis of GATA4 and GATA6 knockdown in AGS and MKN45 cells. Table S7: Differential expression analysis of GATA knockdown in AGS cells. Table S8: GSEA results from GATA KD RNA-seq analysis. Table S9: Statistical analysis of GATA KD qPCR results. Table S10: Gene ontology results from GATA KD RNA-seq analysis. Table S11: Statistical analysis of phenotypic effects from GATA knockdown in AGS and MKN45 cells. Table S12: Statistical analysis of cell cycle and apoptosis for GATA knockdown in AGS and MKN45 cells. Table S13: Statistical analysis of oxidative stress responses for GATA knockdown in AGS and MKN45. Table S14: Regulatory score analysis and selection of GATA-affected targets. Table S15: ROC AUC analysis of staged gastric tumors compared to normal biopsies.
