# Supplementary material for: Deciphering the Landscape of GATA-Mediated Transcriptional Regulation in Gastric Cancer
Source: Antioxidants (Basel). 2024 Oct 18;13(10):1267. doi: 10.3390/antiox13101267 (PMC11504088; doi:10.3390/antiox13101267)
Supplement: Supplementary file 1 [file antioxidants-13-01267-s001.zip › Supplementary Figures/Supplementary Figures antioxidants-3200755.pdf]

Appendix A : Supplementary Figures 1-7 Antioxidants-3200755

Supplementary Figure 1 (related to Figure 2)

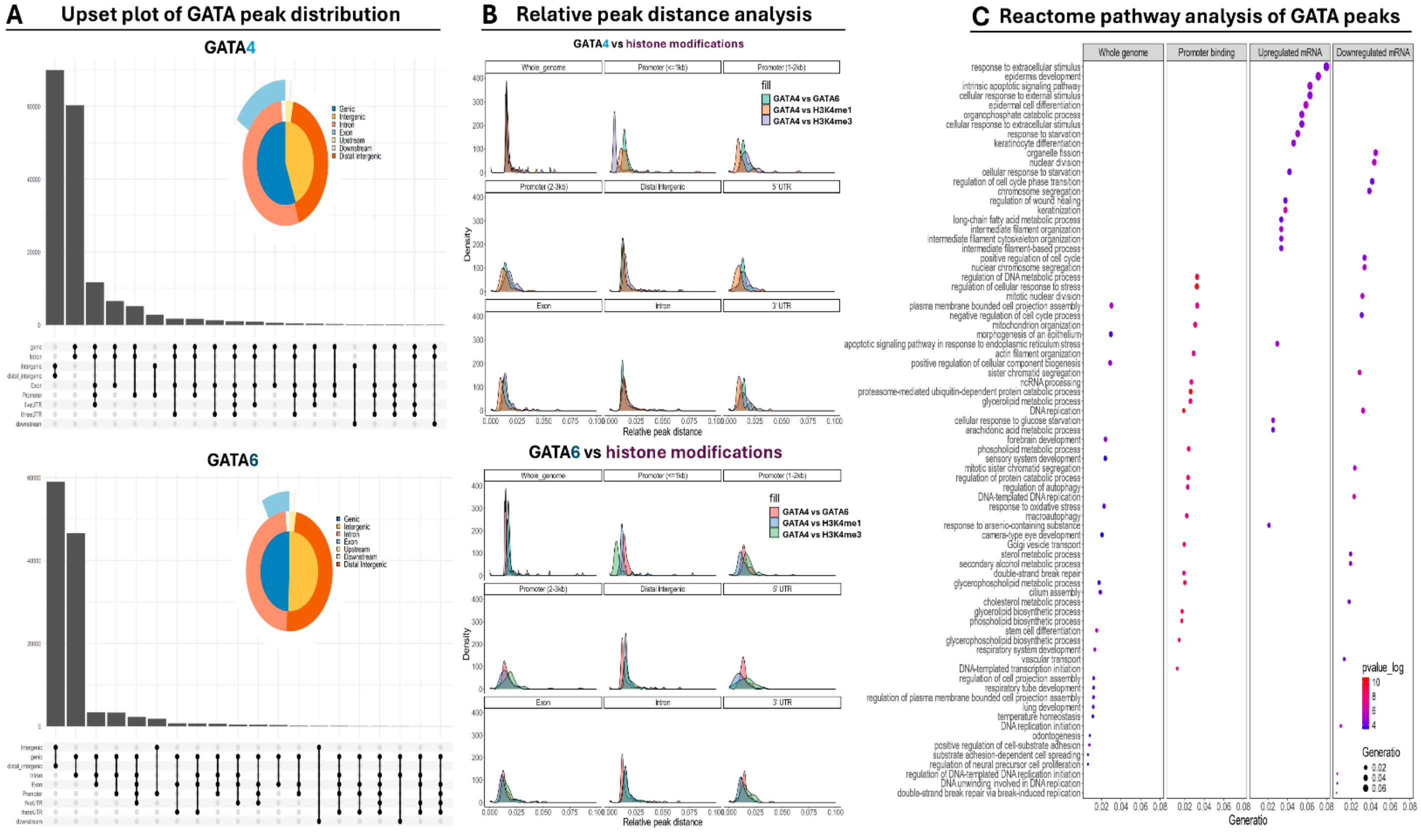

Supplementary Figure S1 : ChIP-seq peak distribution, association and functional analysis in AGS cells A) *Upset plot summarizing the number of GATA4 (upper) and 6 peaks across all genomic annotations as indicated with the dot plot at the bottom. Pie plot summarizes the distribution percentages* ***B)*** *Density plots highlighting the relative peak distance of the GATA4 (upper panel) or GATA6 (lower panel) peaks against H3K4me1 and H3K4me3 across the whole genome or specific genomic annotations. Distributions approaching zero at the X-axis indicate spatial correlation between the corresponding genes, higher score indicates stochastic association between them.* ***C)*** *Functional analysis highlighting the enrichment of reactome pathways for the annotated GATA peaks across various genomic annotations, indicated with the grey box above each dot plot panel. Color indicates statistical significance, dot size indicates gene ratio for each category.*

Supplementary Figure 2 (related to Figure 3)

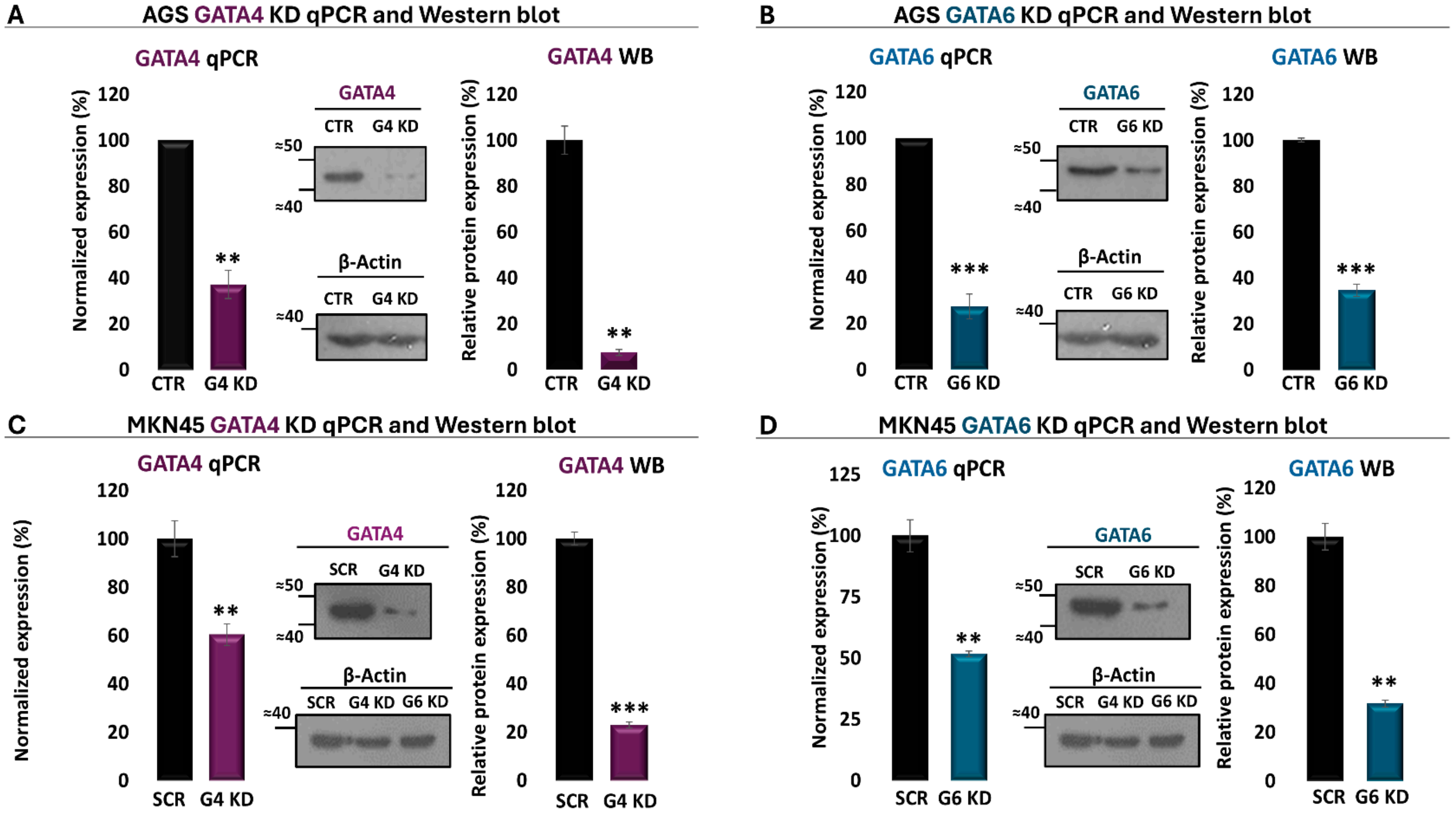

Supplementary Figure S2 : Evaluation of knock-down efficiency for GATA4 and GATA6 in AGS and MKN45 cells A) *RT-qPCR and Western blot analysis for inducible GATA4 KD in AGS cells.* ***B)*** *RT-qPCR and Western blot analysis for inducible GATA6 KD in AGS cells* ***C)*** *RT-qPCR and Western blot analysis for lentiviral mediated knock-down of GATA4 in MKN45 cells* ***D)*** *RT-qPCR and Western blot analysis for lentiviral mediated knock-down of GATA6 in MKN45 cells. * : p-value ⩽0.05, ** : p-value ⩽0.01, ** : p-value ⩽0.001.*

Supplementary Figure 3 (related to Figure 3)

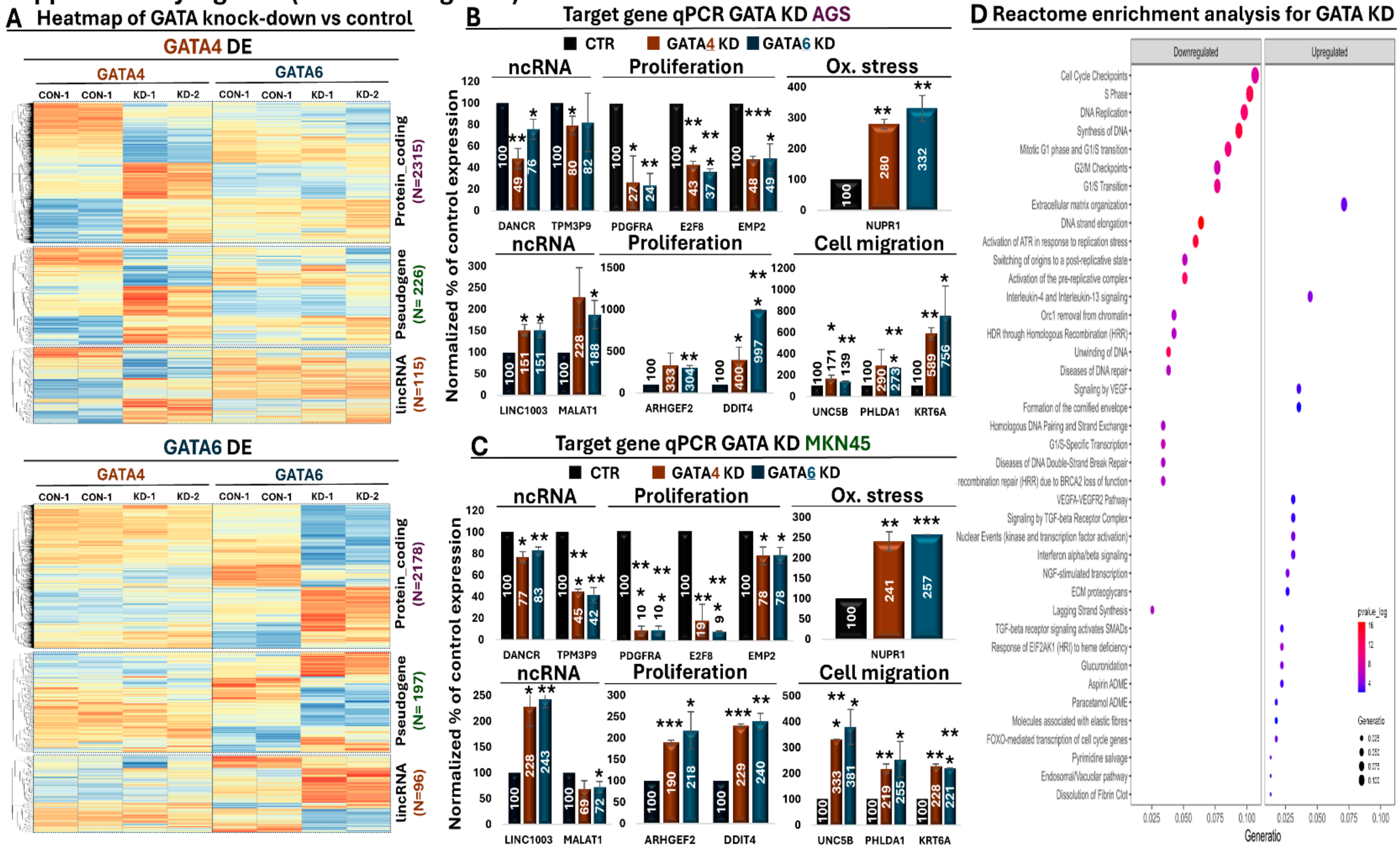

Supplementary Figure S3 : RNA-seq data analysis of GATA KD in gastric cancer cells A) *Heatmap summarizing the normalized expression (z-score) of GATA4 (upper panel) or GATA6 (lower panel) targets across all samples* ***B)*** *qPCR validation of commonly affected GATA target expression in AGS cells (upper panel). Target genes are grouped according to their functional role, indicated above each plot.* ***C)*** *Same as (**B**) for MKN45 cells* ***D)*** *Dot plot summarizing the functional enrichment (reactome pathways) of the up- and down-regulated DEGs upon GATA KD. Color indicates statistical significance, dot size indicates gene ratio for each category. * : p-value $\leqslant$ 0.05, ** : p-value $\leqslant$ 0.01, ** : p-value $\leqslant$ 0.001.*

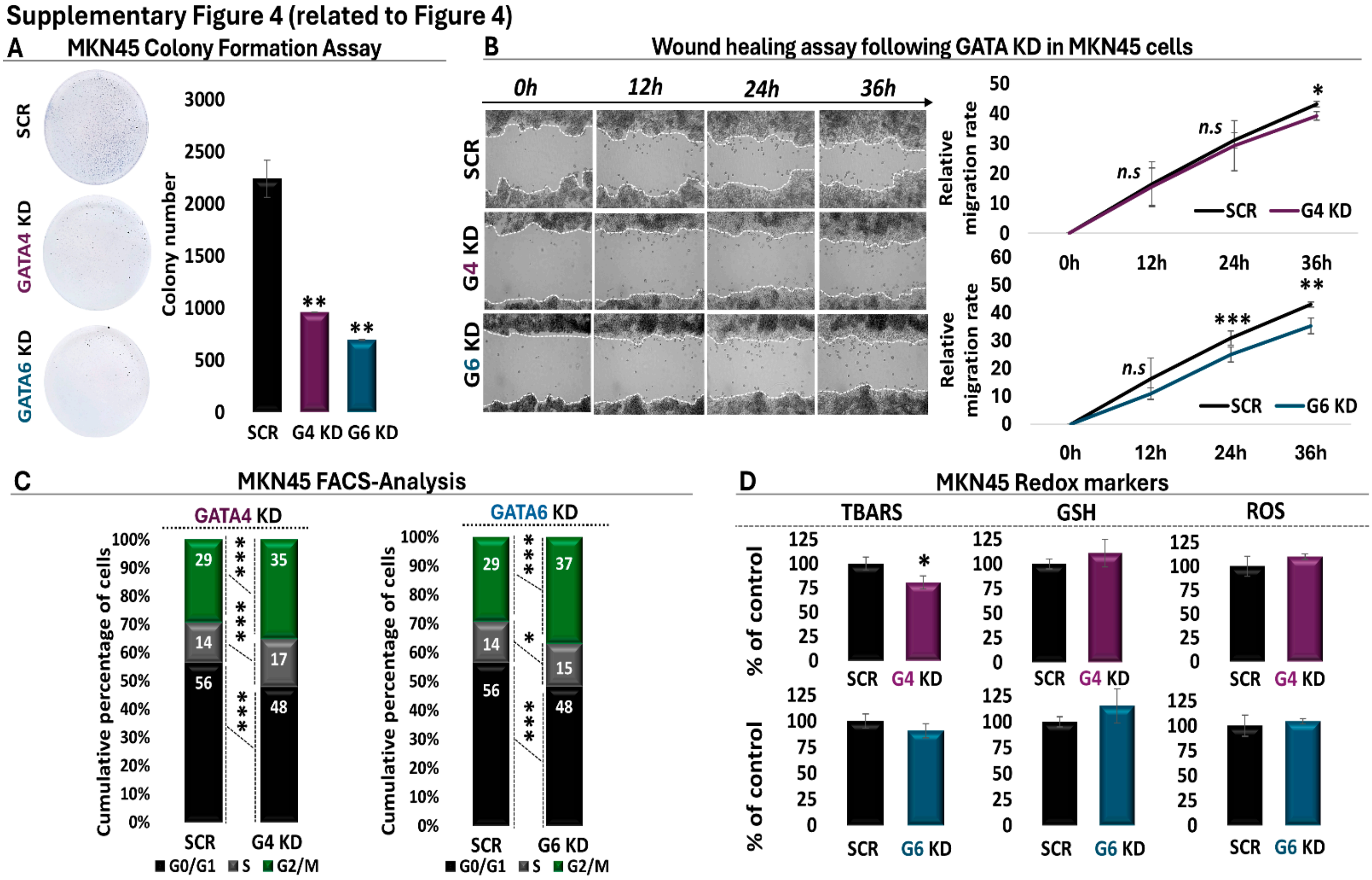

Supplementary Figure S4 : Phenotypic analysis of GATA4 and 6 KD in MKN45 cells A) *Colony formation assay of GATA4 and 6 KD compared to scrambled shRNA control in MKN45 cells. Colony numbers are illustrated as barplots. B) Wound healing assay following GATA4 and 6 KD in MKN45 cells for 0h, 12h, 24h, and 36h post shRNA induction. Graphs illustrate the relative to scrambled control migration index C) FACS cell cycle profile of GATA4 and 6 KD in MKN45 cells D) Redox marker analysis of GATA4 and 6 KD in MKN45 cells. * : p-value ⩽ 0.05, ** : p-value ⩽ 0.01, ** : p-value ⩽ 0.001.*

Supplementary Figure 5 (related to Figure 4)

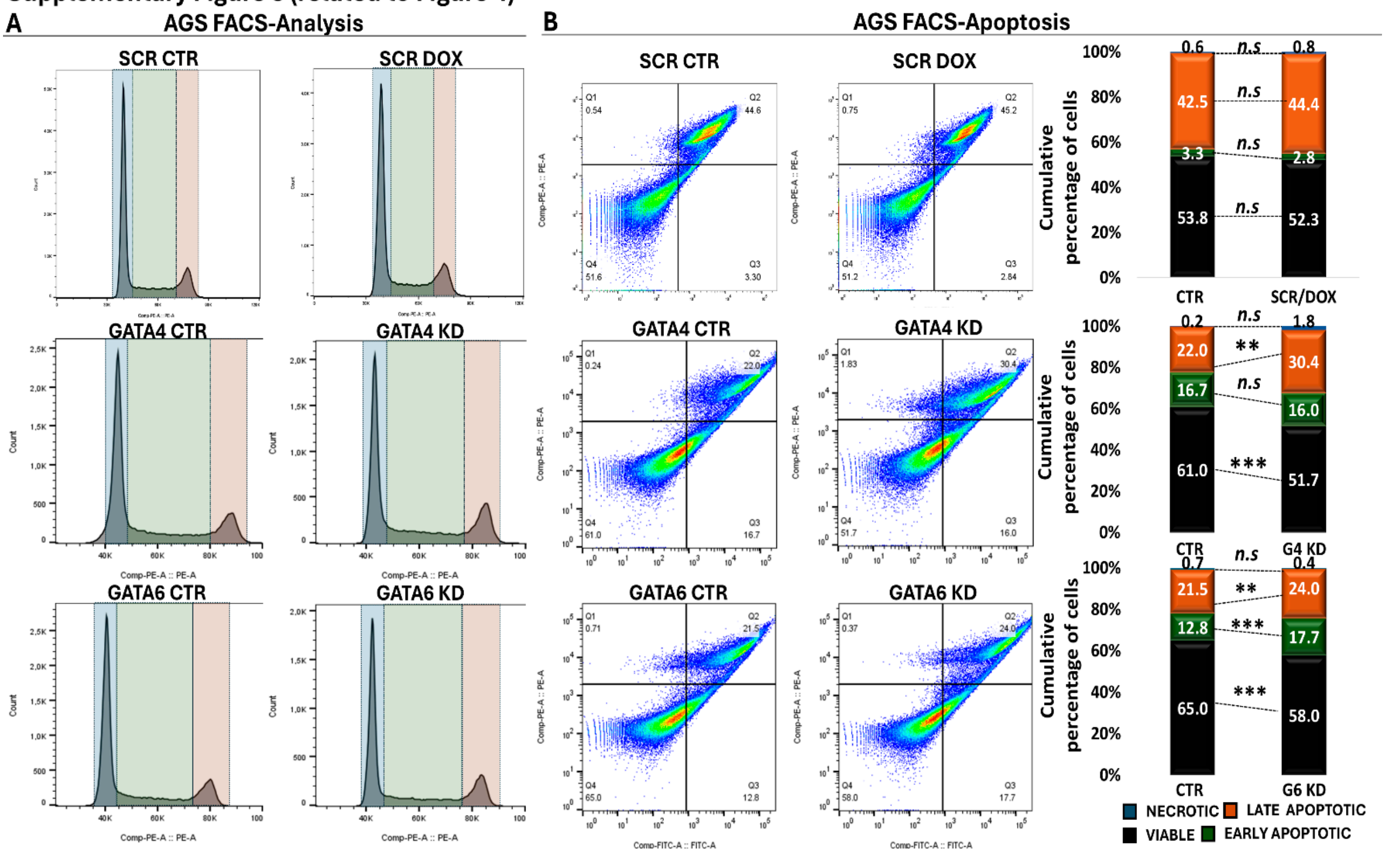

Supplementary Figure S5 : FACS analysis of GATA KD effects on cell cycle and apoptosis in AGS cells A) *Representative cell cycle profile (PI)**,** indicating the distribution (y-axis) of AGS gastric cancer cells in G1/G0 (blue) S (green) and G2/M (red) phase in scrambled shRNA control (upper plot), GATA4 KD (middle plot) or GATA6 KD (bottom plot). Percentages are summarized in Figure 4.* ***B)*** *Apoptosis (Annexin-PI) FACS analysis highlighting the percentage of viable (bottom left quartile, Q4), necrotic (upper left Q1 quartile), late apoptotic (upper right, Q2 quartile) or early apoptotic (bottom right quartile, Q3) for scrambled control (upper plot) GATA4 KD (middle plot) or GATA6 KD (bottom plot). Percentages along with statistical significance are summarized in the stacked bar. * : p-value ⩽ 0.05, ** : p-value ⩽ 0.01, ** : p-value ⩽ 0.001*

Supplementary Figure 6 (related to Figure 4)

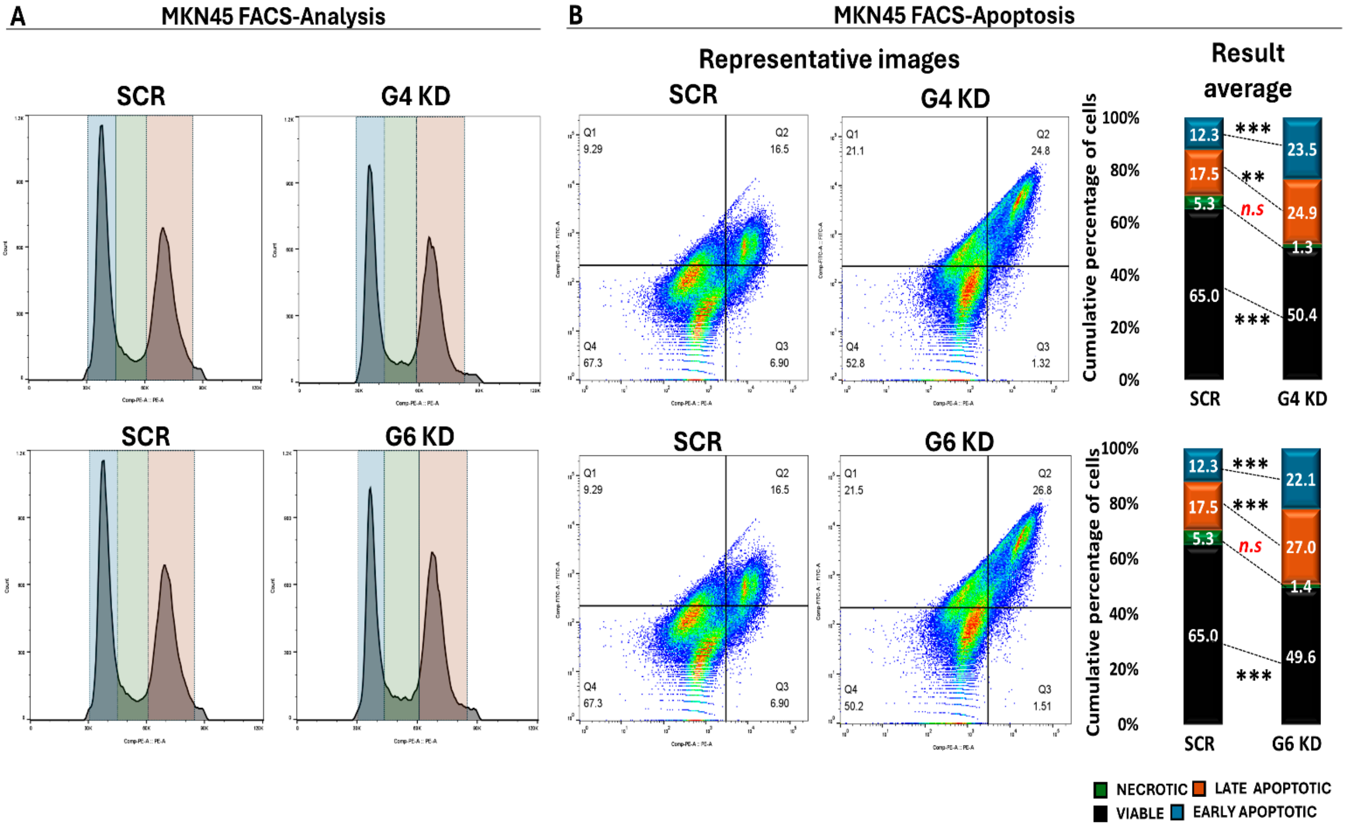

Supplementary Figure S6 : FACS analysis of GATA KD effects on cell cycle and apoptosis in MKN45 cells A) *Representative cell cycle profile (PI)****,*** *indicating the distribution (y-axis) of MKN45 gastric cancer cells in G1/G0 (blue) S (green) and G2/M (red) phase in scrambled shRNA control vs GATA4 KD (upper plot), or GATA6 KD (bottom plot). Percentages are summarized in Supplementary Figure S4.* ***B)*** *Apoptosis (Annexin-PI) FACS analysis highlighting the percentage of viable (bottom left quartile, Q4), necrotic (bottom right Q3 quartile), late apoptotic (upper right, Q2 quartile) or early apoptotic (upper left quartile, Q1) for scrambled control vs GATA4 KD (upper plot) or GATA6 KD (bottom plot). Percentages along with statistical significance are summarized in the stacked bar. * : p-value ⩽ 0.05, ** : p-value ⩽ 0.01, ** : p-value ⩽ 0.001*

Supplementary Figure 7 (related to Figure 5)

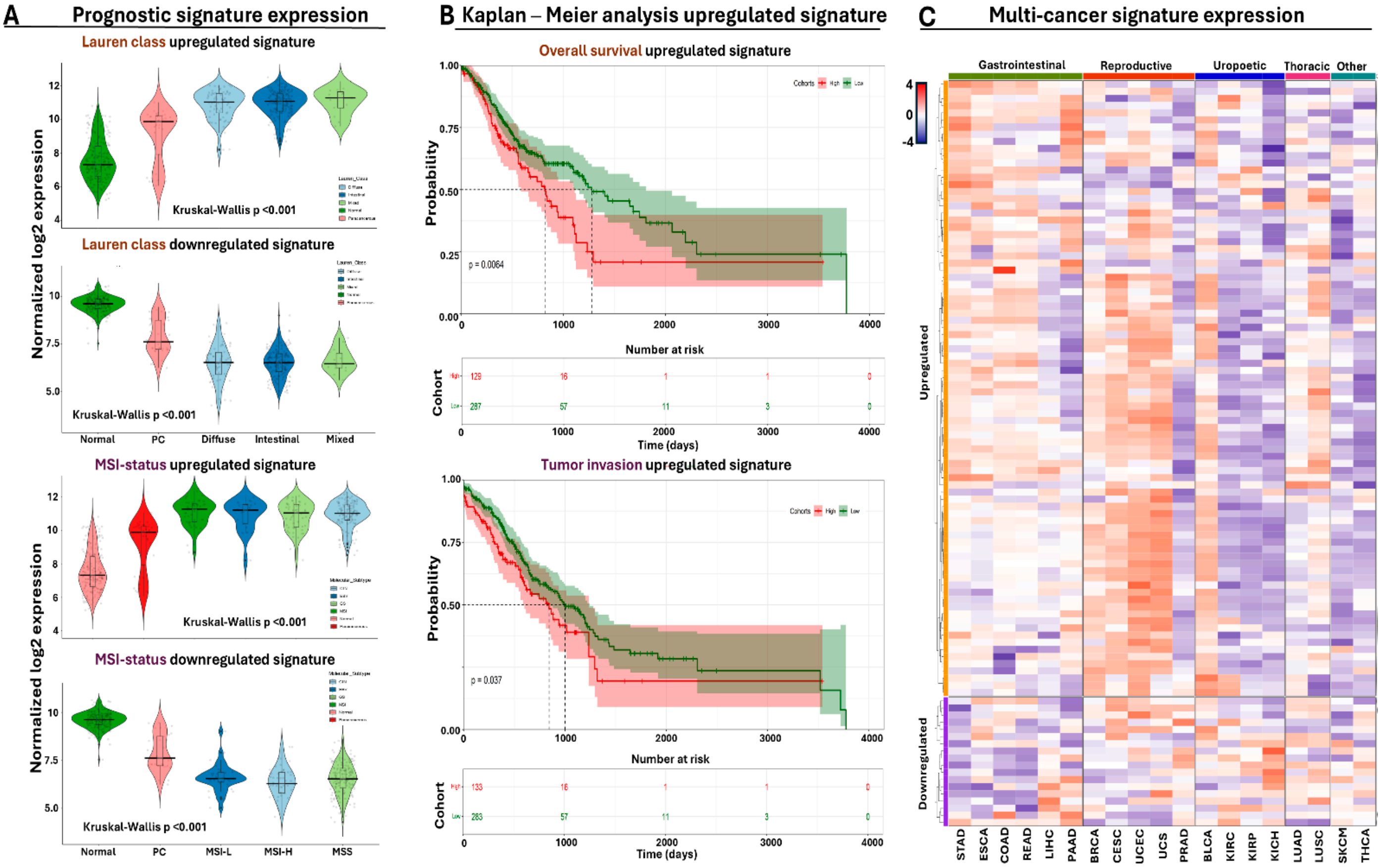

Supplementary Figure S7 : Prognostic potential of meta-signature expression A) *Box plot summarizing the expression of the up- and down-regulated metasignatures across gastric tumors stratified according to Lauren histological classification (upper panel) or MSI score (lower panel) Statistical significance (Kruskal- Wallis) corresponds to normal vs rest comparisons* ***B)*** *Kaplan-Meier analysis for overall survival (upper plot) or lymph node invasion (lower plot) in association with higher (red) or lower (green) expression of the up-regulated meta-signature in gastric tumors C) Heatmap summarizing the differential expression (shown as z-scores of log2 fold change between normal and tumor samples for each cancer type) of the complete panel of 104 GATA-regulated metagenes across 19 cancer types, organized according to system origins (top annotation bars). Cancer type abbreviations are shown [here](#).*
