# Supplementary figures and images for: Deciphering the Landscape of GATA-Mediated Transcriptional Regulation in Gastric Cancer
Source: Antioxidants (Basel). 2024 Oct 18;13(10):1267. doi: 10.3390/antiox13101267 (PMC11504088; doi:10.3390/antiox13101267)

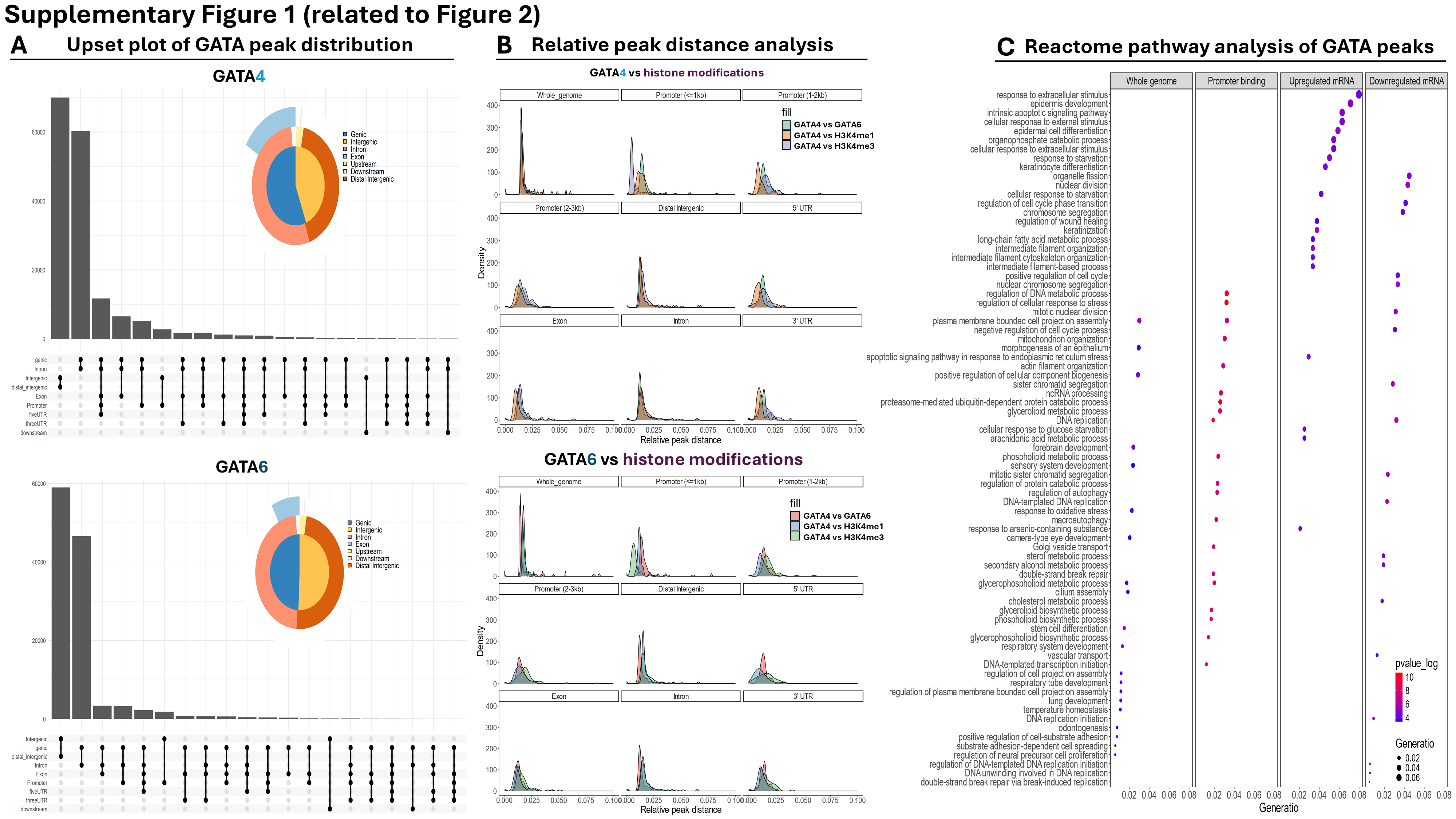

Supplement: Supplementary file 1 [file antioxidants-13-01267-s001.zip › Supplementary Figures/Supplementary Figure 1.TIF]

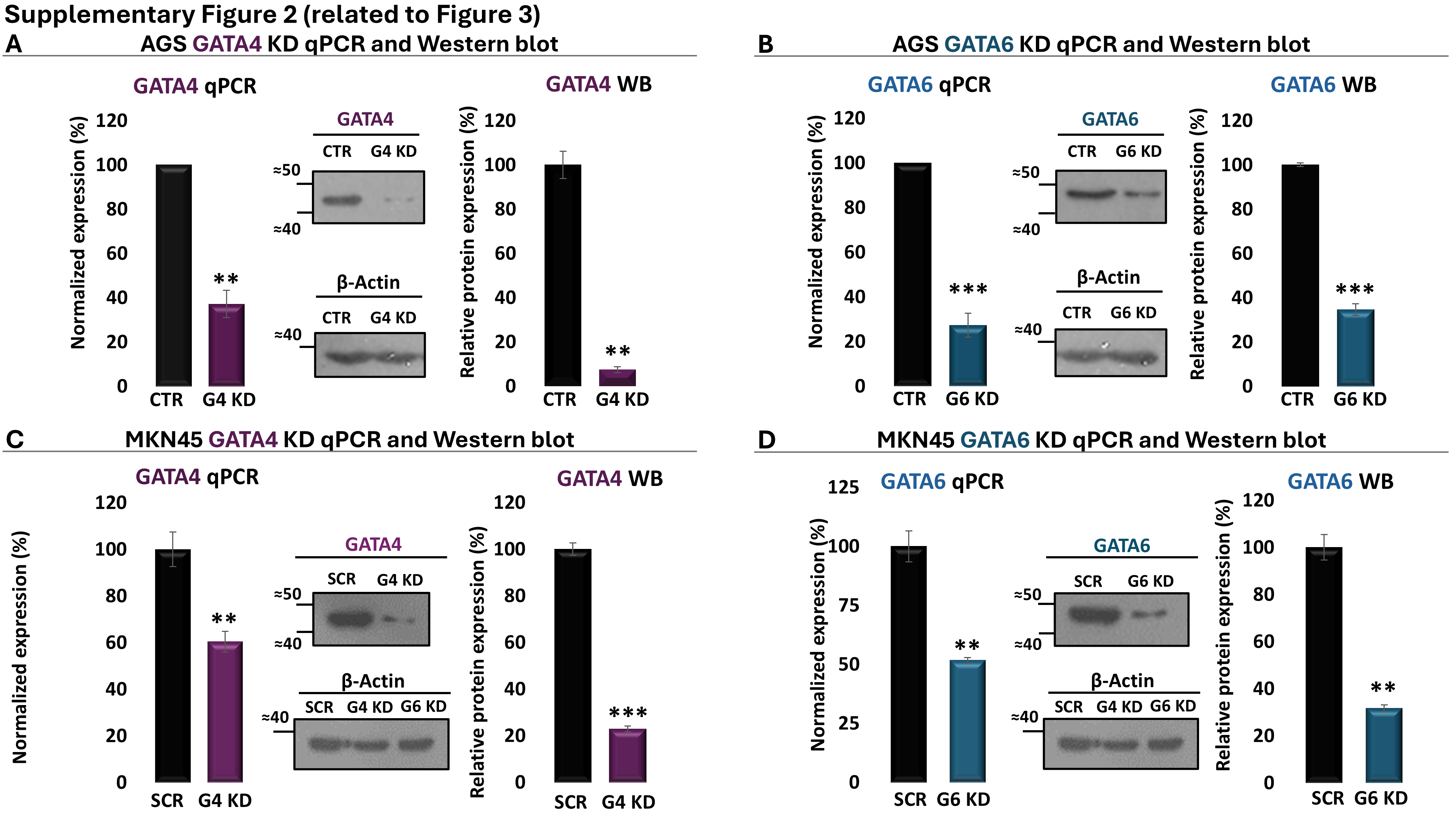

Supplement: Supplementary file 1 [file antioxidants-13-01267-s001.zip › Supplementary Figures/Supplementary Figure 2.TIF]

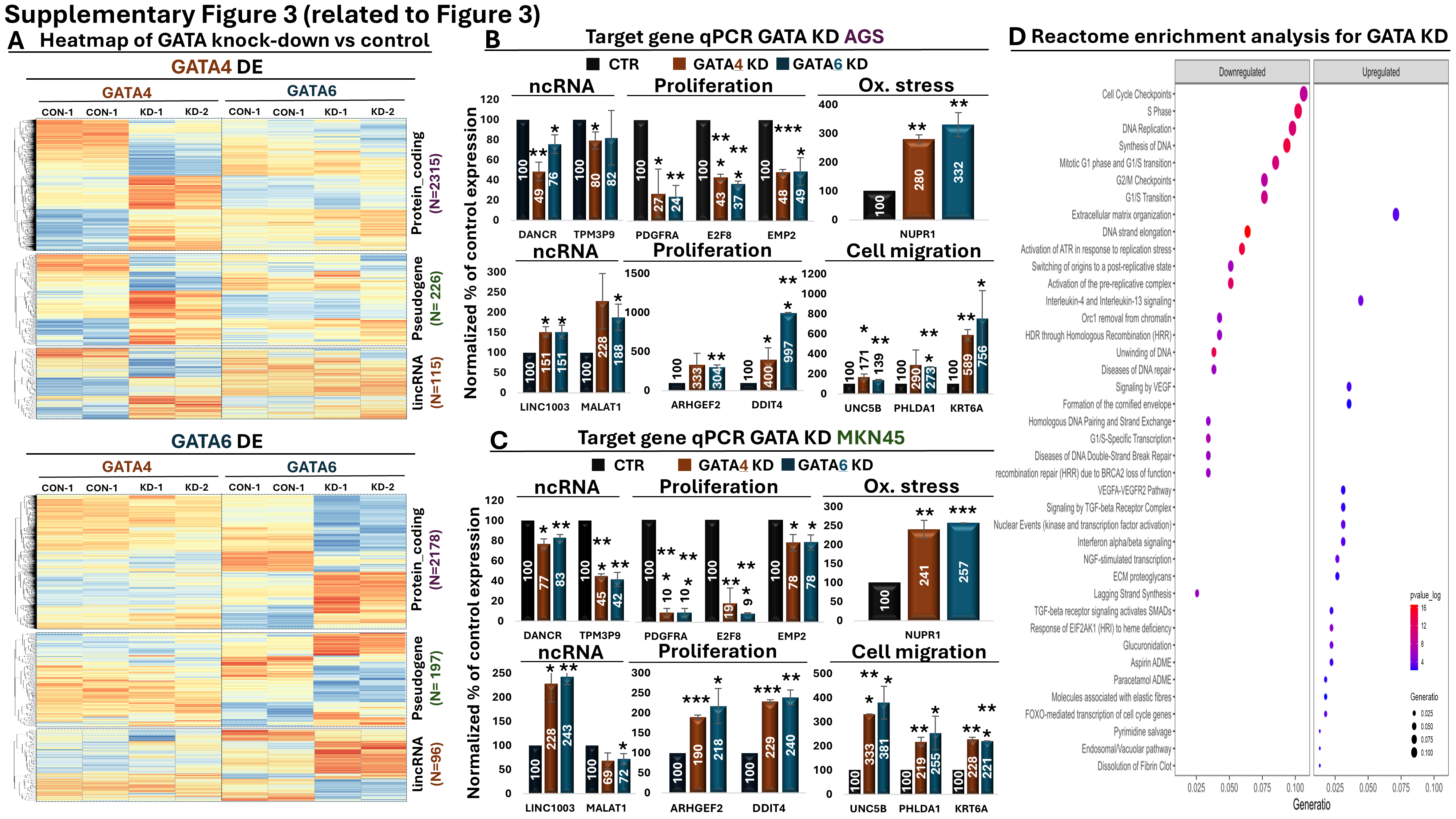

Supplement: Supplementary file 1 [file antioxidants-13-01267-s001.zip › Supplementary Figures/Supplementary Figure 3.TIF]

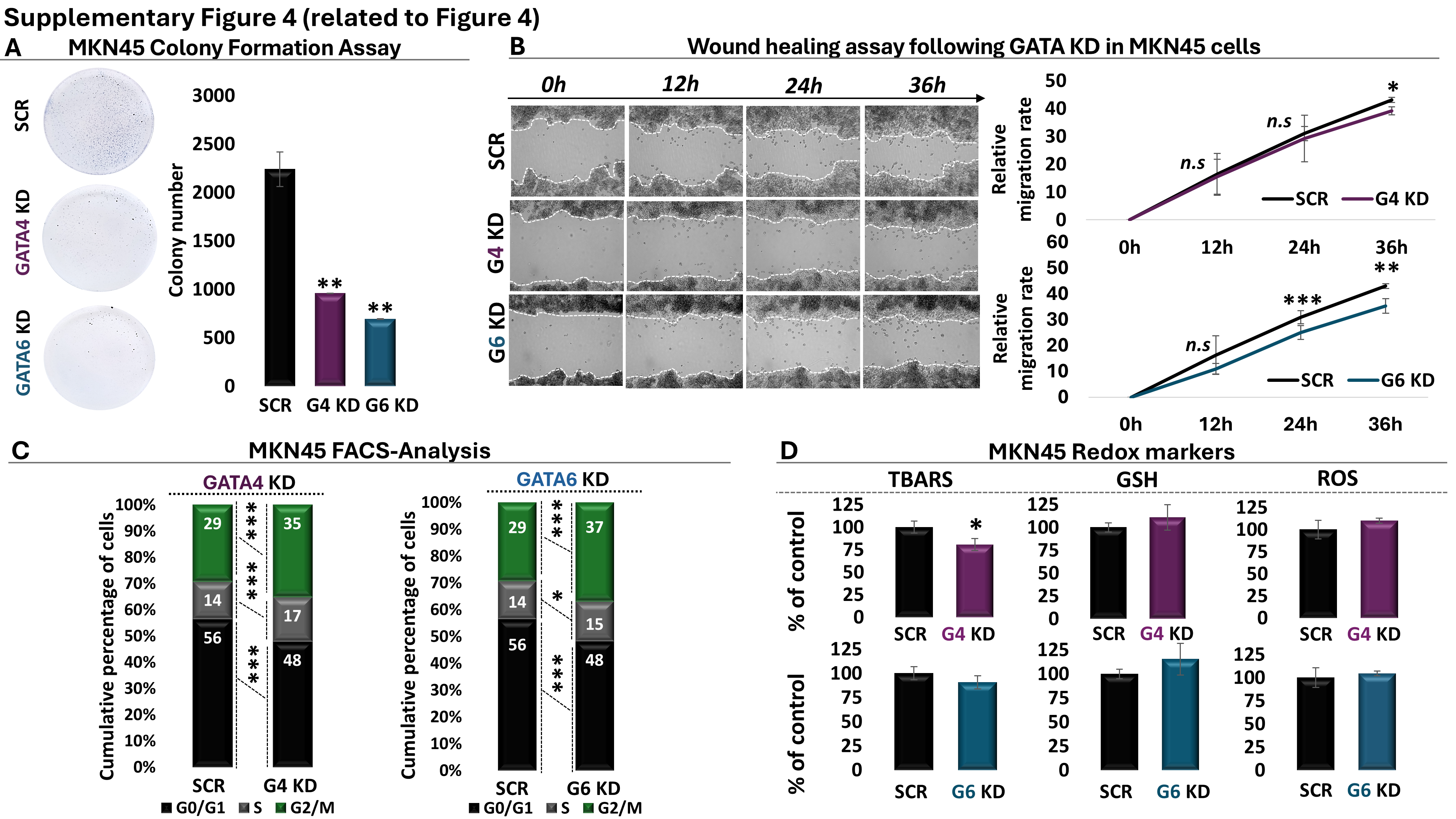

Supplement: Supplementary file 1 [file antioxidants-13-01267-s001.zip › Supplementary Figures/Supplementary Figure 4.TIF]

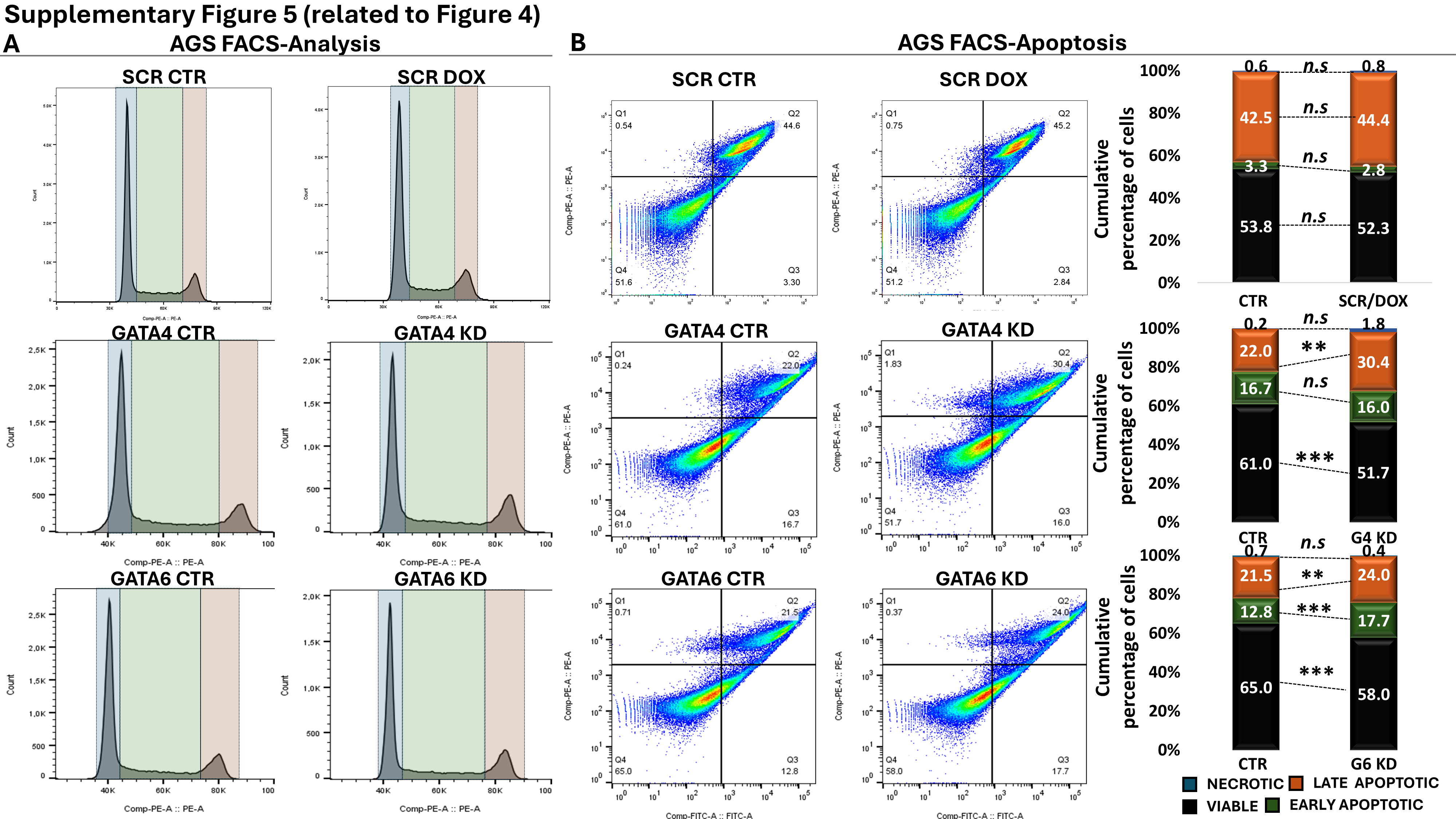

Supplement: Supplementary file 1 [file antioxidants-13-01267-s001.zip › Supplementary Figures/Supplementary Figure 5.TIF]

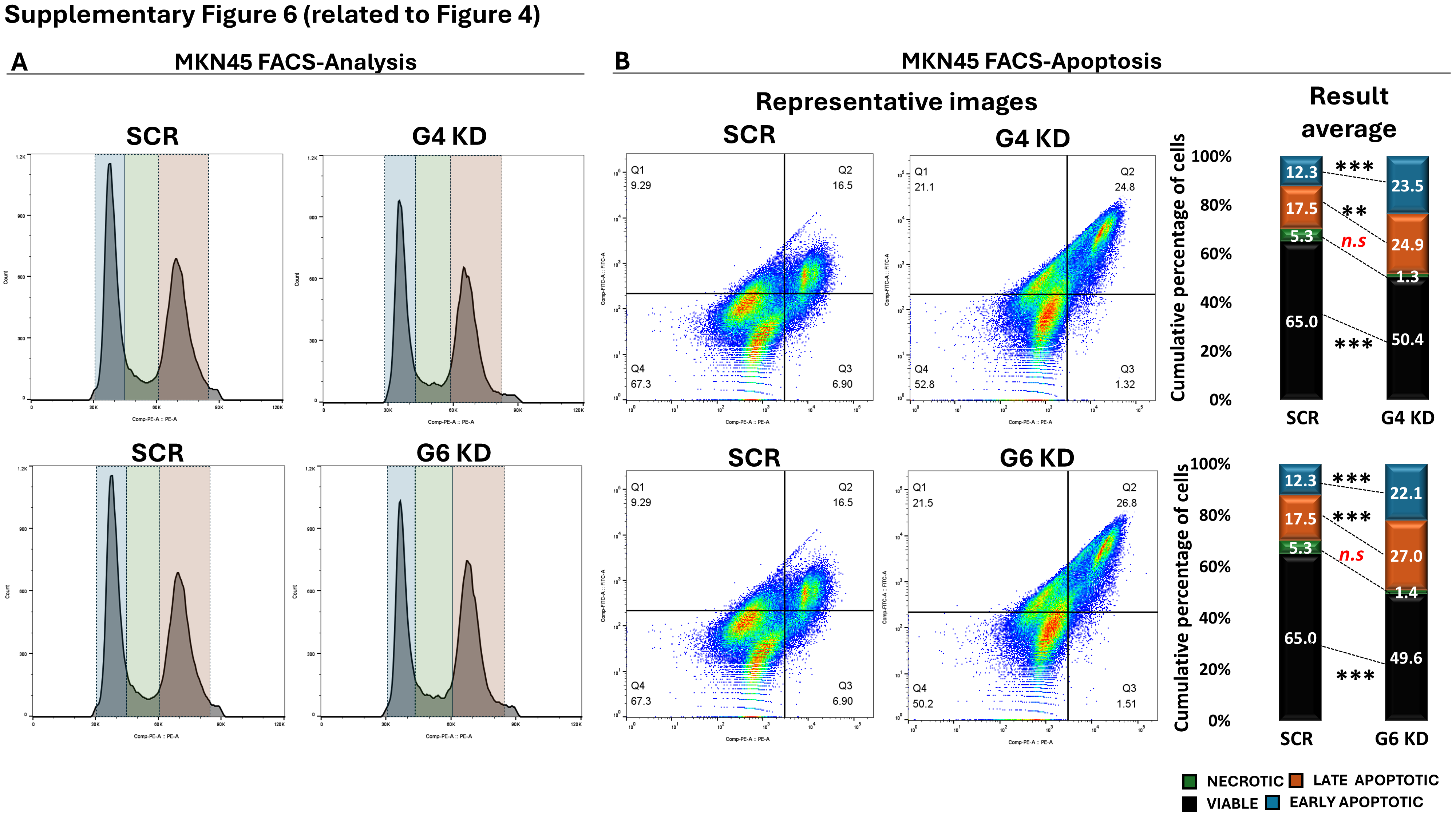

Supplement: Supplementary file 1 [file antioxidants-13-01267-s001.zip › Supplementary Figures/Supplementary Figure 6.TIF]

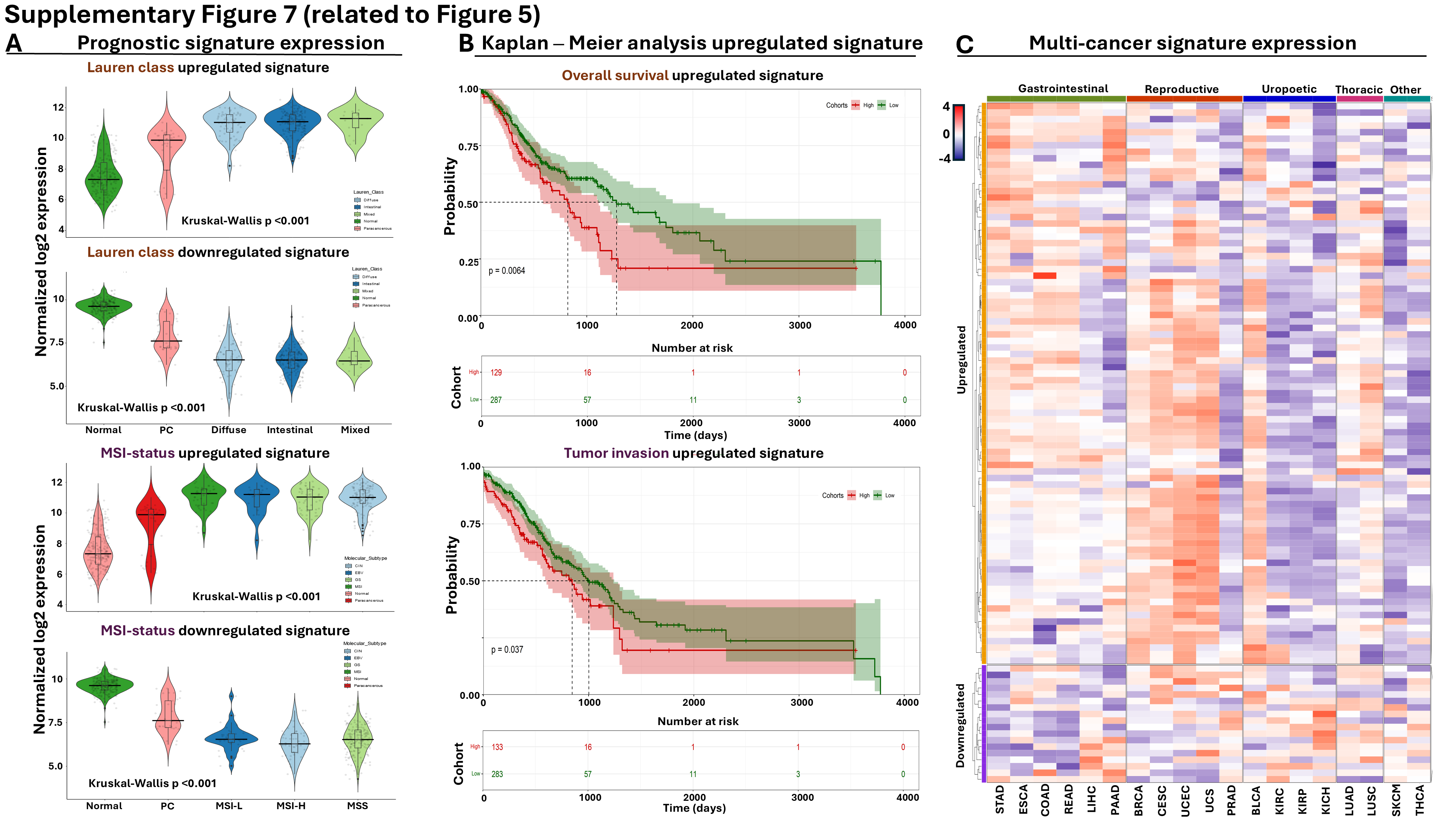

Supplement: Supplementary file 1 [file antioxidants-13-01267-s001.zip › Supplementary Figures/Supplementary Figure 7.TIF]
